# An introduction to new robust linear and monotonic correlation coefficients

**DOI:** 10.1186/s12859-021-04098-4

**Published:** 2021-03-31

**Authors:** Mohammad Tabatabai, Stephanie Bailey, Zoran Bursac, Habib Tabatabai, Derek Wilus, Karan P. Singh

**Affiliations:** 1 Meharry Medical College, Nashville, TN 37208 USA; 2 Department of Biostatistics, Florida International University, Miami, FL 33199 USA; 3 Department of Civil and Environmental Engineering, University of Wisconsin Milwaukee, Milwaukee, WI 53211 USA; 4 Department of Epidemiology and Biostatistics, University of Texas Health Sciences Center at Tyler, Tyler, TX 75708 USA

**Keywords:** Pearson correlation, Spearman correlation, Quadrant correlation, Median correlation, Minimum covariance determinant correlation, Dissimilarity measures, Gene expression, Williams syndrome

## Abstract

**Background:**

The most common measure of association between two continuous variables is the Pearson correlation (Maronna et al. in Safari an OMC. Robust statistics, 2019. https://login.proxy.bib.uottawa.ca/login?url=https://learning.oreilly.com/library/view/-/9781119214687/?ar&orpq&email=^u). When outliers are present, Pearson does not accurately measure association and robust measures are needed. This article introduces three new robust measures of correlation: Taba (T), TabWil (TW), and TabWil rank (TWR). The correlation estimators T and TW measure a linear association between two continuous or ordinal variables; whereas TWR measures a monotonic association. The robustness of these proposed measures in comparison with Pearson (P), Spearman (S), Quadrant (Q), Median (M), and Minimum Covariance Determinant (MCD) are examined through simulation. Taba distance is used to analyze genes, and statistical tests were used to identify those genes most significantly associated with Williams Syndrome (WS).

**Results:**

Based on the root mean square error (RMSE) and bias, the three proposed correlation measures are highly competitive when compared to classical measures such as P and S as well as robust measures such as Q, M, and MCD. Our findings indicate TBL2 was the most significant gene among patients diagnosed with WS and had the most significant reduction in gene expression level when compared with control (*P *value = 6.37E-05).

**Conclusions:**

Overall, when the distribution is bivariate Log-Normal or bivariate Weibull, TWR performs best in terms of bias and T performs best with respect to RMSE. Under the Normal distribution, MCD performs well with respect to bias and RMSE; but TW, TWR, T, S, and P correlations were in close proximity. The identification of TBL2 may serve as a diagnostic tool for WS patients. A Taba R package has been developed and is available for use to perform all necessary computations for the proposed methods.

**Supplementary Information:**

The online version contains supplementary material available at 10.1186/s12859-021-04098-4.

## Background

Novel measures of correlation that have noticeably improved performance over existing measures can be a fundamental enhancement to understanding data, affecting a broad range of fields. One of the most widely used statistical measures is the correlation coefficient. The choice of correlation and dissimilarity measures is essential in many areas of science including, but not limited to, clustering co-expressed genes, mediation and moderation analysis with structural equation modeling, time series analysis, pattern recognition, autonomous robots, structural engineering, image recognition, graph theoretical algorithms, spatiotemporal trajectory, artificial intelligence, machine learning techniques, classification, principal component analysis, discriminant analysis, and correlation graphs [1–12]. The need for robust techniques is of utmost significance when dealing with high dimensional biological noisy data. Biological bioassay data frequently contain outliers [13]. Therefore, the choice of the metric can considerably affect the analysis results.

Various resistant dissimilarity measures, such as Tukey's biweight estimate proposed by Hardin et al., are available in the literature, however Pearson (P), Spearman (S), and Euclidian dissimilarity measures are the most commonly used techniques in biomedical research [14, 15]. For standardized vectors $X$ and $Y$ with dimensions $n$, the Euclidean distance ${d}_{Euclid}$ is related to Pearson distance ${d}_{Pearson}$ [16] by the following equation:

$$d_{Euclid} = 2\sqrt {n*d_{Pearson} } .$$

The choice of distance measure to assess outliers plays a vital role in determining the outcome of a wide range of applications [17].

A major difficulty in clustering large data is in the usage of an appropriate dissimilarity measure that captures the geometrical characteristics of those data [18]. Shevlyakov and Pavel Smirnov examined the robustness of correlation coefficient estimators under the assumption of normality at various sample sizes [19]. In a simulation study, Winter et al. concluded that the P correlation coefficient is appropriate when the underlying distribution is light-tailed; but, if outliers are present or the underlying distribution is heavy-tailed, then S correlation coefficient should be used [20]. Using a centroid based algorithm, Shirkhorshidi et al. concluded that P correlation performs well at high dimensions but not in low dimensions [21]. Robust correlation was identified as a more useful tool in image-guided surgery applications and image registration in radiotherapy [22].

Pearson dissimilarity measure has frequently been used in the assessment of cell-lines using expression levels or sequence variation profiles genome-wide [23]. Yona et al. studied the quality of some dissimilarity measures used in microarray analysis in order to determine the most effective measure(s) for detecting functional links [24]. A robust complementary hierarchical clustering was introduced to guard against genes with outlying expression levels [25]. Moore et al. utilized the correlation coefficient to examine the association between the quality of visually graded chest images and a quantitative assessment of chest phantom images [26]. Several other studies integrated clever bias-reducing techniques such as drawing from the Weibull distribution in analysis, creating new dissimilarity measures with a normalization factor, and testing the performance of logistic and conventional probabilistic hidden variable models when dealing with gene expression data [27–29], they claimed that these methods helped to mitigate the negative effects of outliers from the data.

The role of DNA methylation in regulating the expression of oncogenes and progression of cancer types also has been found to generate many outliers. A robust correlation coefficient is a vital tool for calculating the correlation between DNA methylation and gene expression in epigenetic studies when outliers are present [30, 31]. The use of an improper correlation can result in a variety of patterns that produce conflicting results regarding gene expression [30]. Nishimura et al. assessed whether the volume of infused crystalloid fluid is correlated to the amount of interstitial fluid leakage during surgery [32], and Kim et al. studied whether opioid growth factor receptor expression is correlated with cell proliferation in cancer cells [33].

Bloch et al. found that improvement in gene clustering can be obtained by applying the Median correlation measure when outliers are present [34]. The choice of dissimilarity measure is essential part of the RNA transcriptome data analysis, which can determine similar genes or tissues, leading to the identification of biomarkers of specific diseases and the discovery of new drug interventions [35].

Our simulation results indicate that in the presence of outliers or influential observations, non-robust correlation measures of dissimilarity often result in conclusions that do not represent the true association. We have developed robust linear and monotonic correlation measures capable of giving an accurate estimate of correlation when outliers are present, and reliable estimates when outliers are absent. In this paper, Taba (T), TabWil (TW), and TabWil rank (TWR) correlations are introduced and their robustness are validated by a simulation study in comparison with other widely used correlation estimators.

## Methods

### Definition 1

The function ${T}_{\omega }$: ${\varvec{R}} \rightarrow {\varvec{R}}$ is defined as ${T}_{\omega }(x)=x*\mathrm{Sech}(\upomega *x)$, where Sech is the hyperbolic secant function and $\upomega$ is the tuning constant. ${T}_{\omega }$ has the following properties:

- $${T}_{\omega }(0) = 0$$
- For every real number $x$, ${T}_{\omega }(-x)$ = $-{T}_{\omega }(x)$
- For every nonnegative real number $x$, ${T}_{\omega }$(x) ≥ 0
- $$\frac{\mathrm{d}({T}_{\omega }(\mathrm{x}))}{\mathrm{d}(\mathrm{x})}$$ = 1 when $$x$$ = 0
- $${T}_{\omega }(x$$) $$\rightarrow$$ 0 as $$|x|$$ $$\rightarrow \infty$$
- $$\frac{\mathrm{d}({T}_{\omega }(\mathrm{x}))}{\mathrm{d}(\mathrm{x})}\rightarrow 0$$ as $$|x|$$ $$\rightarrow\infty$$
- For every positive real number $k,$ $\frac{{T_{\omega } \left( {kx} \right)}}{{T_{\omega } \left( x \right)}}$ $= k$ as $x$ $\rightarrow 0$
- $$T_{\omega } \left( x \right)$$ is bounded

**Fig. 1:**
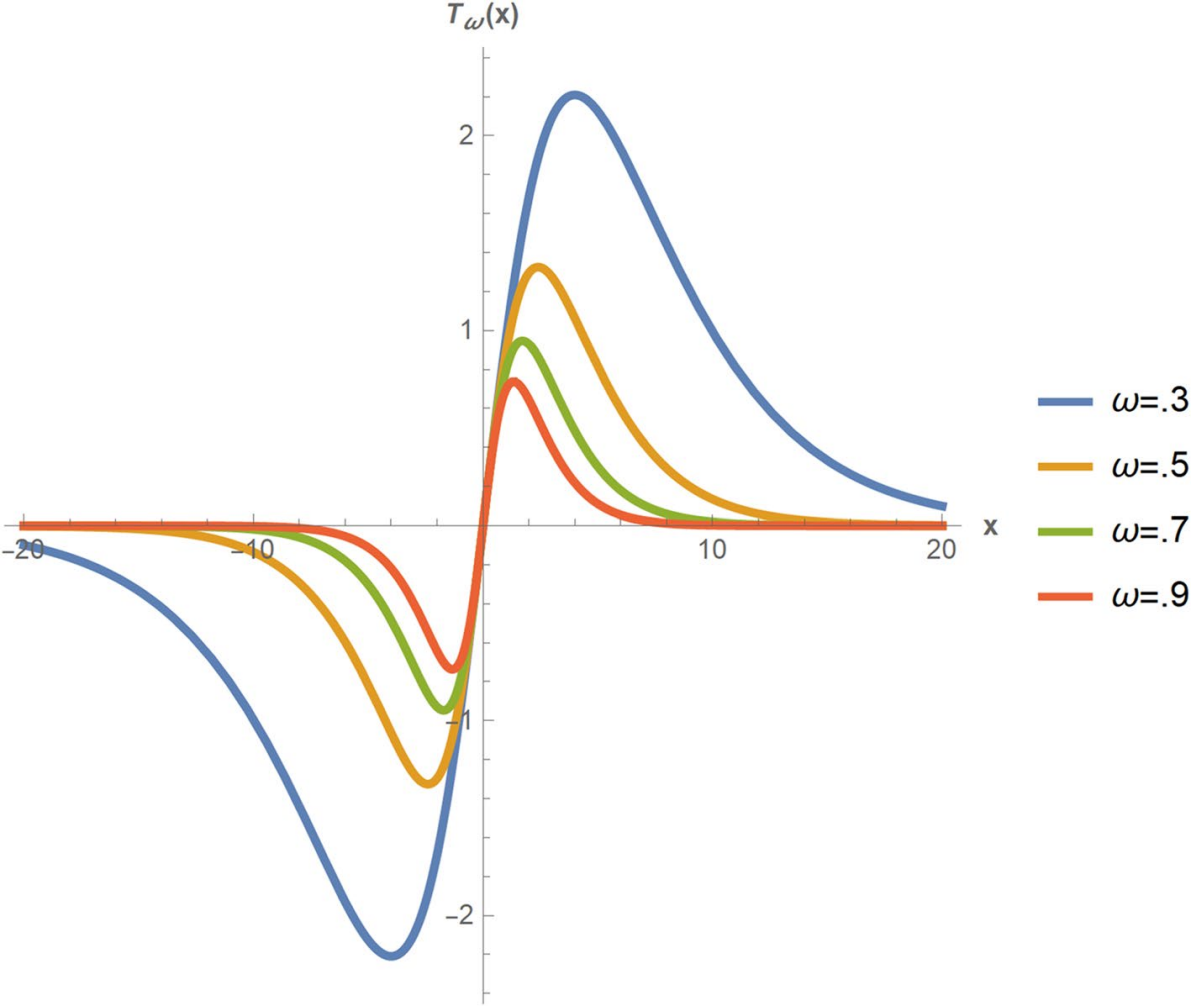
Graph of $T_{\omega } \left( x \right)$ using Wolfram Mathematica software version 12.1

Figure 1 depicts the function $T_{\omega } \left( x \right)$ for various values of ${\upomega }$, illustrating the properties mentioned in Definition 1. The value of ${\upomega }$ has been calculated using asymptotic efficiency under the assumption of normality [36, 37]. The re-descending property of the function can be seen as $\left| x \right|$ approaches infinity. The function $T_{\omega }$ is a bounded influence function. Due to its properties mentioned in Definition 1, our proposed measures of correlation have high efficiency, a high breakdown point, and will not suffer from masking effects [38, 39].

## Robust correlation methods

### Taba correlation

For variables $X$ and $Y$ each of size $n,$ we define the Taba robust correlation coefficient $r_{Taba}$ as:

$$r_{Taba} \left( {X,Y} \right) = \frac{{\mathop \sum \nolimits_{i = 1}^{n} \left[ {T_{\omega } \left( {C_{1,i} } \right)*T_{\omega } \left( {C_{2,i} } \right)} \right]}}{{\sqrt {\mathop \sum \nolimits_{i = 1}^{n} \left[ {T_{\omega } \left( {C_{1,i} } \right)} \right]^{2} *\mathop \sum \nolimits_{i = 1}^{n} \left[ {T_{\omega } \left( {C_{2,i} } \right)} \right]^{2} } }} ,$$

where $C_{1,i} = \frac{{x_{i} - Median\left( X \right)}}{{{\hat{\sigma }}_{{S_{n} \left( X \right)}} }}$, $C_{2,i} = \frac{{y_{i} - Median\left( Y \right)}}{{{\hat{\sigma }}_{{S_{n} \left( Y \right)}} }}.$ Dispersion measures ${\hat{\sigma }}_{{S_{n} \left( X \right)}}$ and ${\hat{\sigma }}_{{S_{n} \left( Y \right)}}$ are estimates of the standard deviation for variables $X$ and $Y$ respectively, introduced by Rousseeuw and Croux as a robust scale measurement. Other robust choices such as ${\hat{\sigma }}_{{Q_{n} \left( \cdot \right)}}$ are available as an alternative estimate of standard deviation [40, 41]. For the Taba correlation estimator, we set our default value for $\omega$ at 0.45 which will give us over 95% in asymptotic efficiency under normality assumptions [36].

### TabWil correlation

Let $$U = \frac{X - Median\left( X \right)}{{{\hat{\sigma }}_{{S_{n} \left( X \right)}} }} + \frac{Y - Median\left( Y \right)}{{{\hat{\sigma }}_{{S_{n} \left( Y \right)}} }}$$ and $$V = \frac{X - Median\left( X \right)}{{{\hat{\sigma }}_{{S_{n} \left( X \right)}} }} - \frac{Y - Median\left( Y \right)}{{{\hat{\sigma }}_{{S_{n} \left( Y \right)}} }}$$.

**Fig. 2:**
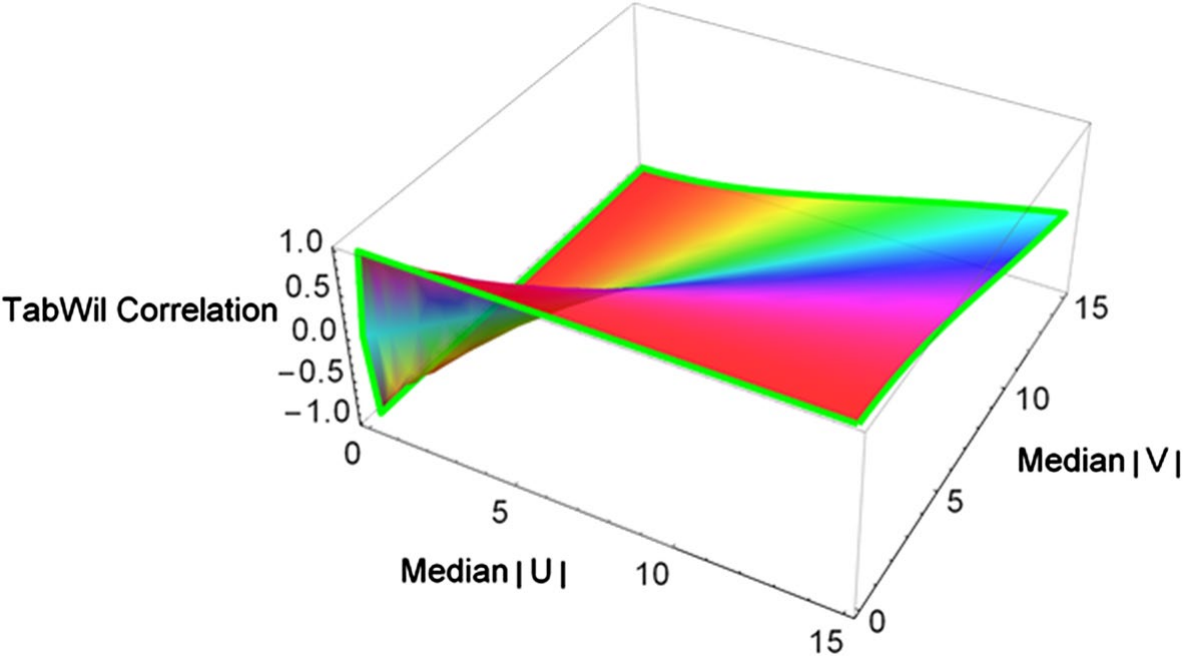
Graph of TabWil Correlation using Wolfram Mathematica software version 12.1

We define the robust TabWil correlation estimator $r_{TabWil}$ as:

$$r_{TabWil} \left( {X,Y} \right) = \frac{{T_{\omega } \left( {m_{1}^{2} } \right) - T_{\omega } \left( {m_{2}^{2} } \right)}}{{T_{\omega } \left( {m_{1}^{2} } \right) + T_{\omega } \left( {m_{2}^{2} } \right)}} ,$$

where $m_{1} = Median\left( {\left| U \right|{ }} \right)$ and $m_{2} = Median\left( {\left| V \right|{ }} \right).$ Both T and TW correlations estimate the linear association between variables $X$ and $Y$. Figure 2 illustrates the TW correlation coefficient as a function of $Median\left( {\left| U \right|{ }} \right)$ and $Median\left( {\left| V \right|{ }} \right)$.

### TabWil rank correlation

For vectors $X$ and $Y$, let $R_{X} = Rank\left( X \right)$, $R_{Y} = Rank\left( Y \right)$, where $Rank\left( X \right)$ and $Rank\left( Y \right)$ refer to the ordinal standing of each element in the vectors $X$ and $Y,$ respectively.

Define

$$D_{1} = \frac{{R_{X} - Median\left( {R_{X} } \right)}}{{{\hat{\sigma }}_{{S_{n} \left( {R_{X} } \right)}} }} + \frac{{R_{Y} - Median\left( {R_{Y} } \right)}}{{{\hat{\sigma }}_{{S_{n} \left( {R_{Y} } \right)}} }}\quad {\text{and}}\quad D_{2} = \frac{{R_{X} - Median\left( {R_{X} } \right)}}{{{\hat{\sigma }}_{{S_{n} \left( {R_{X} } \right)}} }} - \frac{{R_{Y} - Median\left( {R_{Y} } \right)}}{{{\hat{\sigma }}_{{S_{n} \left( {R_{Y} } \right)}} }}$$

then the robust TabWil rank correlation estimator $r_{TabWilRank}$ is defined as:

$$r_{TabWilRank} \left( {X,Y} \right) = \frac{{T_{\omega } \left( {L_{1}^{2} } \right) - T_{\omega } \left( {L_{2}^{2} } \right)}}{{T_{\omega } \left( {L_{1}^{2} } \right) + T_{\omega } \left( {L_{2}^{2} } \right)}} ,$$

where $L_{1} = Median\left( {\left| {D_{1} } \right|{ }} \right)$ and $L_{2} = Median\left( {\left| {D_{2} } \right|{ }} \right)$ and the default value for $\omega$ is 0.05 for both TW and TWR correlations. These values were determined using asymptotic efficiency and outlier tolerance using simulation. There is a trade-off between asymptotic efficiency and outlier tolerance level. In other words, the lower the efficiency, the higher the tolerance level [37, 42].

The TWR correlation estimator measures the monotonic association and direction between two variables $X$ and $Y$. The TWR correlation can be used with ordinal, interval, or ratio data.

### Confidence intervals for proposed Measures

The $\left( {1 - \alpha } \right)100 \%$ confidence interval estimator for correlation $\rho$ using any of the three proposed robust measures ($r_{\left( \cdot \right)}$) is given by the following lower ($LCL_{\left( \cdot \right)}$) and upper ($UCL_{\left( \cdot \right)}$) confidence limits:

$$LCL_{\left( \cdot \right)} = Tanh\left( {F_{\left( \cdot \right)} { } - Z_{{1 - \frac{\alpha }{2}}} \sqrt {\frac{{1 + 0.5r_{\left( \cdot \right)}^{2} }}{{\left( {n - 3} \right)}}} } \right)$$

and

$$UCL_{\left( \cdot \right)} = Tanh\left( {F_{\left( \cdot \right)} + Z_{{1 - \frac{\alpha }{2}}} \sqrt {\frac{{1 + 0.5r_{\left( \cdot \right)}^{2} }}{{\left( {n - 3} \right)}}} } \right),$$

where $F_{\left( \cdot \right)} = ArcTanh\left( {r_{\left( \cdot \right)} } \right)$ is the Fisher transformation of robust correlation measure $r_{\left( \cdot \right)} .$ The symbols $Tanh\left( \cdot \right)$ and $ArcTanh\left( \cdot \right)$ represent the hyperbolic tangent and inverse hyperbolic tangent functions respectively. Bonett and Wright as well as Ruscio studied confidence intervals constructed using $\frac{{1 + 0.5r_{\left( \cdot \right)}^{2} }}{{\left( {n - 3} \right)}}$ as an estimate for variance of Fisher transformation. Their results indicate that $\left( {1 - \alpha } \right)100 \%$ confidence interval for $\rho$ provide fairly accurate coverage when a robust correlation measure is used [43, 44]. For one sided confidence limits, simply replace $\frac{\alpha }{2}$ by $\alpha$ in the equation for $LCL_{\left( \cdot \right)}$ or $UCL_{\left( \cdot \right)}$. Alternative methods, such as bootstrapping, are also available for calculating confidence interval estimates [45].

### Testing hypothesis for proposed measures

To test the researcher (alternative) hypotheses $H_{1} :\rho \ne 0$, $H_{1} :\rho > 0$, or $H_{1} :\rho < 0$ using any of the three proposed robust correlation measures $r_{\left( \cdot \right)} ,$ one can utilize the test statistic $t_{{\left( {n - 2} \right)}} = r_{\left( \cdot \right)} \sqrt {\frac{n - 2}{{1 - 0.5r_{\left( \cdot \right)}^{2} }}}$ with $n - 2$ degrees of freedom.

## Simulation study

The aim of our simulation study is to assess the performance of our proposed methods in comparison with other correlation estimators in the presence and absence of outliers. To achieve this aim,

We have used RStudio version 1.3.1073 utilizing lcmix, robustbase, mvtnorm, Taba, robcor, MethylCapSig, and MultiRNG packages to assess the performance of T, TW, and TWR in comparison with P, S, Quadrant (Q), Median (M), and Minimum Covariance Determinant (MCD) correlation estimators [46, 47]. We generated m = 5000 pairs of samples each having size $n$ = 20, 40, 80, 160, or 320 from one of three distributions: a bivariate normal with mean vector $$\left( {\begin{array}{*{20}c} 0 \\ 0 \\ \end{array} } \right),$$ a bivariate log-normal with mean vector $$\left( {\begin{array}{*{20}c} 1 \\ 3 \\ \end{array} } \right),$$ or a bivariate Weibull with a shape parameter of 1.5. All bivariate distributions had a variance–covariance matrix of the form $$\left( {\begin{array}{*{20}c} 1 & {\uprho } \\ {\uprho } & 1 \\ \end{array} } \right),$$ where the five levels of correlation $\rho$ used in our simulation were set at the 0.0, 0.2, 0.5, 0.7, and 0.9 levels. Random contaminations of our simulated data were generated at the 0%, 5%, and 10% levels. To do this, each iteration randomly drew the appropriate number of observations (based on the level of contamination) to be corrupted. For each of the selected datapoints, the contaminated datapoints will be equal to the value of the uncontaminated datapoints plus five times the standard deviation of the uncontaminated sample within each iteration (positive shift). The results were similar with a negative shift, but are not shown here. After contamination, the correlation was calculated using each of the eight correlation methods. T, TW, and TWR correlations used tuning constants $\omega = 0.45, 0.05, {\text{and}} \,0.05$ respectively. For comparative purposes, bias and the root mean square errors (RMSE) were calculated for all methods. The bias and the RMSE are defined as:

$$bias = \left| {\frac{{\mathop \sum \nolimits_{l = 1}^{m} \hat{\rho }_{l} }}{m} - {\uprho }} \right|$$

and

$$RMSE = \sqrt{\frac{{\mathop \sum \nolimits_{l = 1}^{m} (\hat{\rho }_{l} - {\uprho })^{2} }}{m}} .$$

## Simulation results

To better understand our simulation results, we ordered the bias and RMSE for each distribution and identified the correlation estimators associated with the ordered results as shown in Additional file 1: Table S1.

### Simulation results stratified by sample size

**Fig. 3:**
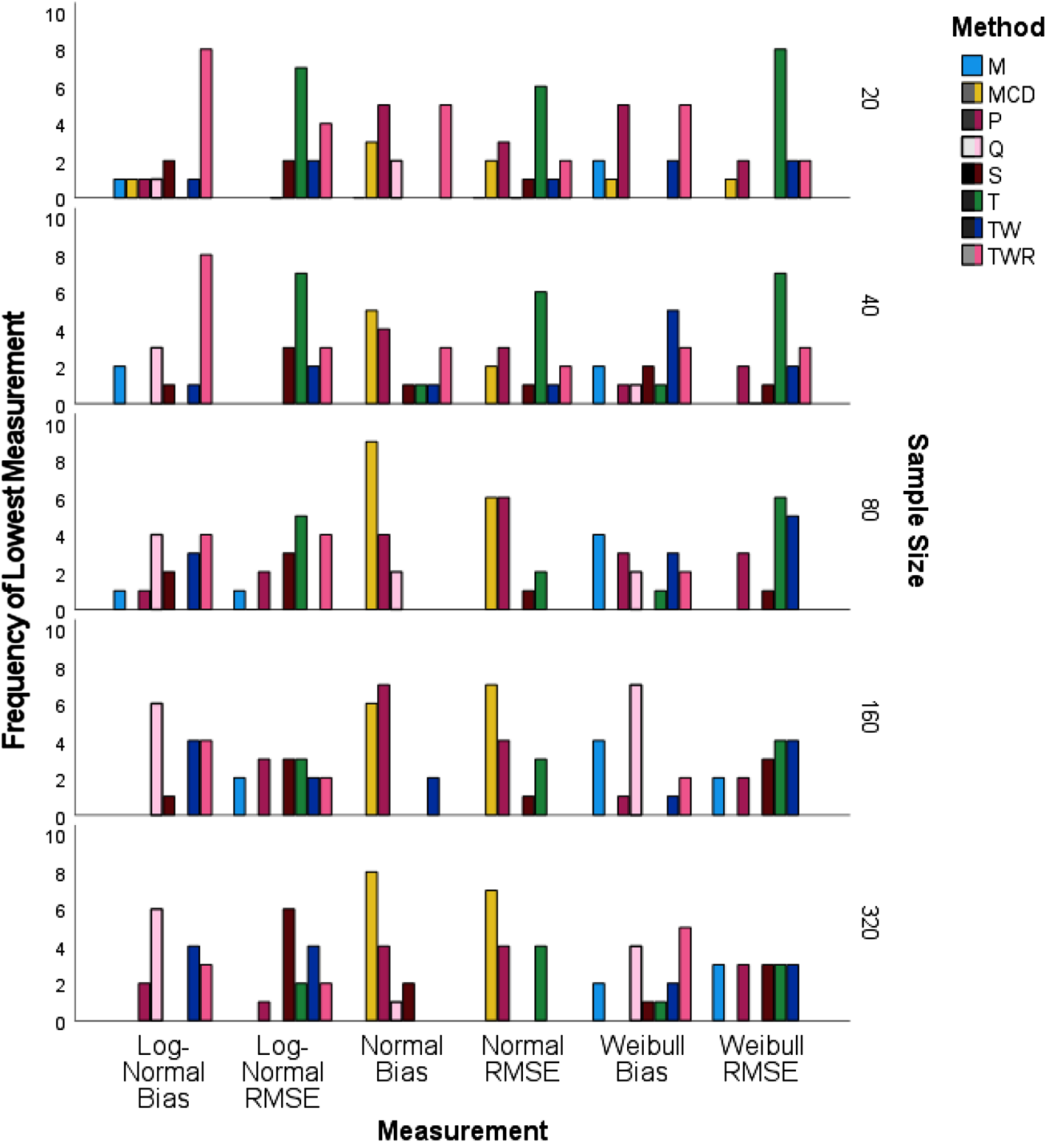
Frequency of lowest measurement for simulated data stratified by sample size using IBM SPSS software version 27

Figure 3 compares the frequency of each correlation method that resulted in having the lowest bias or RMSE in our simulation study, stratified by sample size. For small samples of size 20, TWR or T correlations consistently had the highest frequency of lowest bias and RMSE; tying with P correlation under bias for the bivariate Normal and bivariate Weibull distributions.

For samples of size 40, T correlation uniformly performed best with respect to RMSE under all three bivariate distributions. TWR correlation was shown to have the lowest bias the greatest number of times when the distribution was bivariate Log-Normal, while MCD and TW regularly appeared as having the lowest bias for bivariate Normal and Weibull distributions respectively.

Samples of size 80 or more consistently showed MCD and P correlations having the lowest bias and RMSE under the assumption of a Normal distribution. Assuming the distribution is Weibull, T correlation had the highest frequency of lowest bias or was among methods having the most frequent lowest RMSE. When the distribution was Log-Normal, Q correlation generally had the highest frequency of lowest bias with samples larger than 80, but tied with TWR when the sample size was set to 80. For other non-Normal cases, T correlation performed well with respect to RMSE in Log-Normal distributions, but was overtaken by S and TW correlations when the sample size increased to 320. M correlation performed best with respect to bias when the distribution was bivariate Weibull.

### Simulation results stratified by level of correlation $\left( {{\varvec{\uprho}}} \right)$

**Fig. 4:**
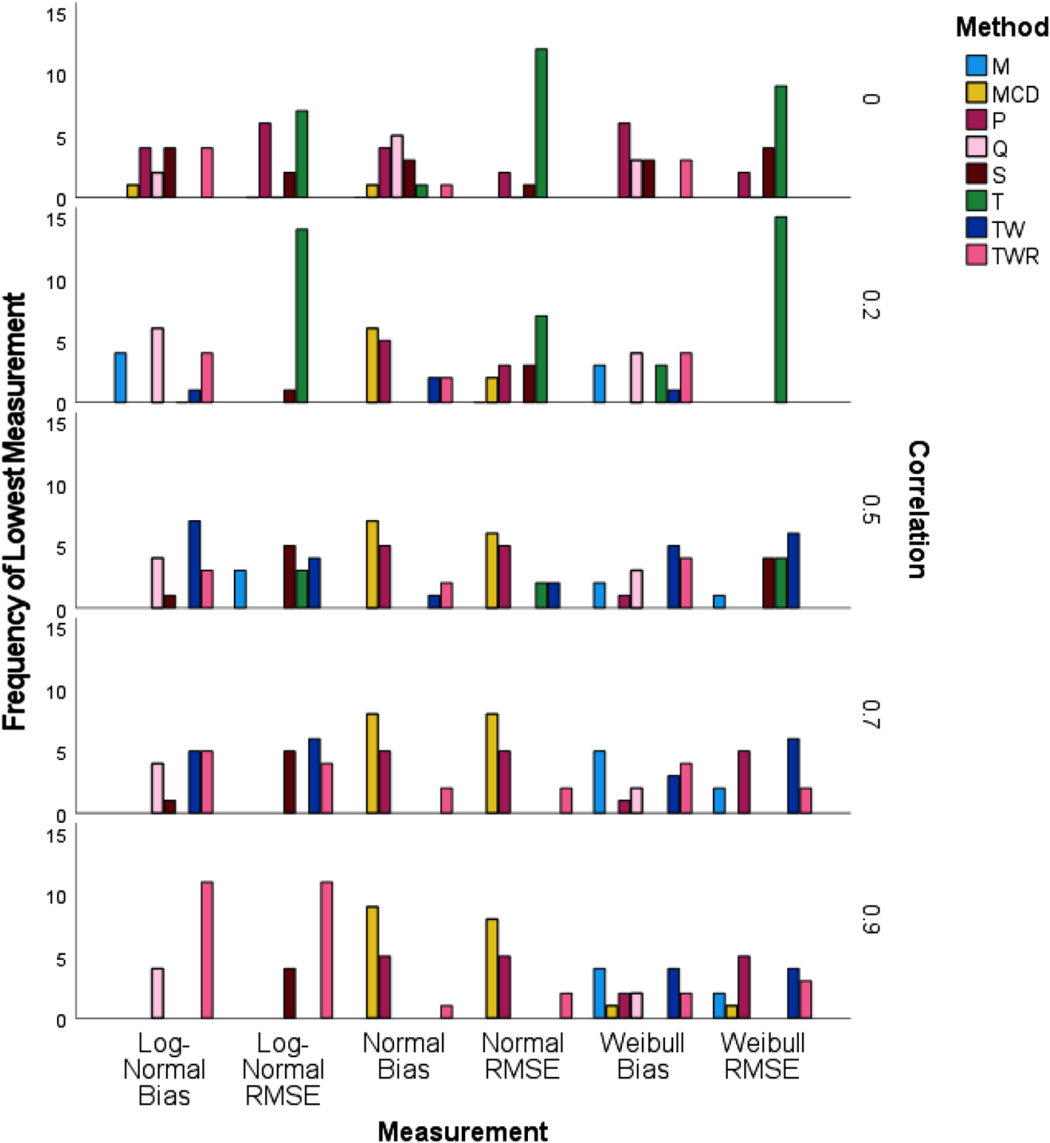
Frequency of lowest measurement for simulated data stratified by simulated value of correlation using IBM SPSS software version 27

Similar to the previous graphic, Fig. 4 depicts the frequency of lowest measurements, this time stratified by the value of correlations. As ${\uprho }$ becomes more positive, the sampling distribution of correlation estimator becomes left skewed. When correlation is set to zero, T correlation performed best in RMSE for all tested bivariate distributions. P correlation performed well overall in terms of bias, but tied with TWR and S correlations when the distribution was bivariate Log-Normal and was overtaken by Q correlation when the distribution was normal.

For correlations set at 0.2, T correlation outperformed the other correlation methods for RMSE in all three bivariate distributions. Q performed best with regard to bias in the bivariate Log-Normal, but the MCD performed best when the distribution was bivariate Normal. TWR and Q tied for the best performance in bias when the distribution was bivariate Weibull.

When the correlation level was raised to 0.5, TW performed best in bias for both bivariate Log-Normal and Weibull. MCD performed best with regard to bias and RMSE for bivariate Normal. S and TW performed best in RMSE for bivariate Log-Normal and Weibull distributions, respectfully.

At the 0.7 correlation level for the bivariate Normal distribution, MCD performed best with regard to bias and RMSE, but TW performed best in RMSE for both bivariate Weibull and bivariate Log-Normal distributions. M performed best with respect to bias in the bivariate Weibull distribution, while TW and TWR tied for the best bias performance in the bivariate Log-Normal.

Finally, when the correlation reached the 0.9 level, the best performance in bias and RMSE for both bivariate Log-Normal and Normal belonged to TWR and MCD respectively. TW gave the best performance in terms of RMSE for the bivariate Weibull; TW and M tied for the best performance in bias for the bivariate Weibull distribution.

### Simulation results stratified by level of contamination

**Fig. 5:**
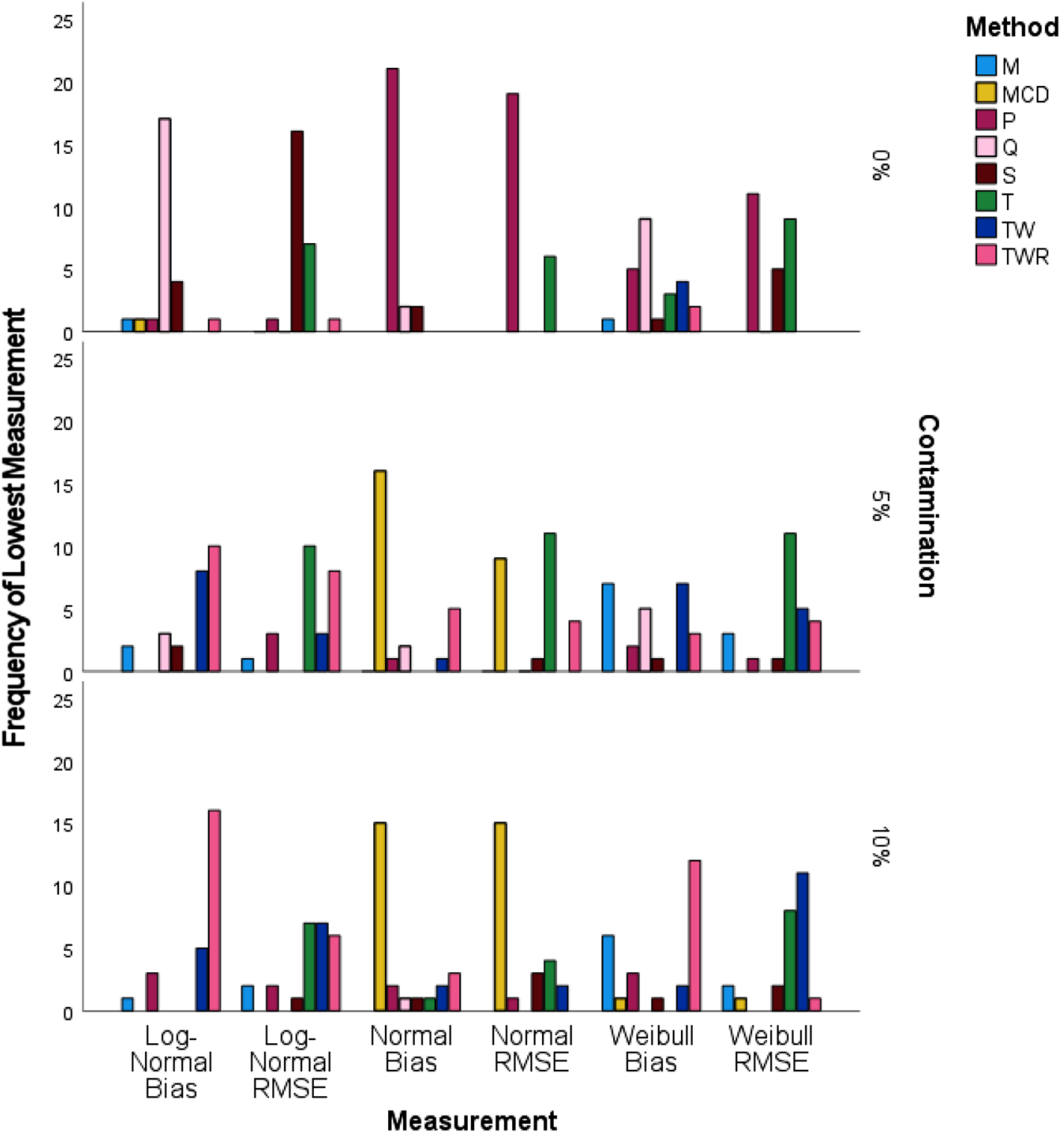
Frequency of lowest measurement for simulated data stratified by contamination level using IBM SPSS software version 27

When the frequency of lowest measurements was stratified by the levels of data contamination, we observed that in the absence of contamination, the best performing bias and RMSE belonged to P correlation. Q correlation had the best performance in bias for both the bivariate Log-Normal and bivariate Weibull distributions. Figure 5 shows that S and P had the best performance with regard to RMSE for bivariate Log-Normal and bivariate Weibull respectively.

At the 5% level of contamination, T had the best performance with regard to RMSE for all three distributions. MCD performed best with regard to bias for the bivariate Normal and TWR had the best performing bias for the bivariate Log-Normal. TW and M tied for the best performance in bias for the bivariate Weibull. Finally, when contamination level reached 10%, MCD performed best in both bias and RMSE for bivariate Normal, while TWR performed best in bias for both the bivariate Log-Normal and bivariate Weibull. TW performed best with respect to RMSE for bivariate Weibull. There was a tie between TW and T for the best performing RMSE when the distribution was bivariate Log-Normal.

### Overall simulation results

**Fig. 6:**
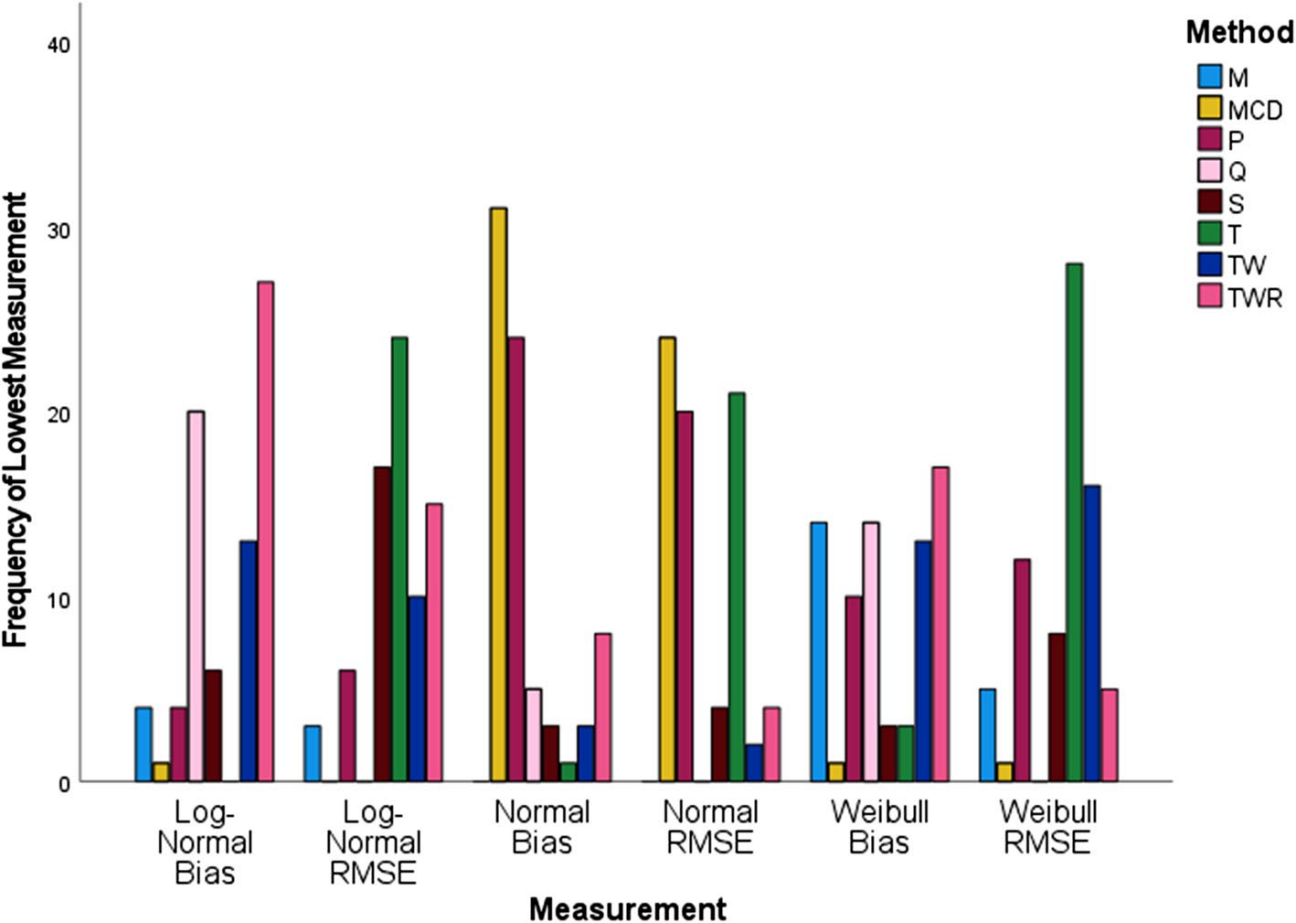
Frequency of lowest measurement for simulated data using IBM SPSS software version 27

Overall, as indicated in Fig. 6, for the bivariate Normal, MCD had the best performance with respect to bias and RMSE, but when the distribution was bivariate Log-Normal or bivariate Weibull, TWR performed best in bias and T had the best performance with respect to RMSE.

## Analysis of William syndrome

**Fig. 7:**
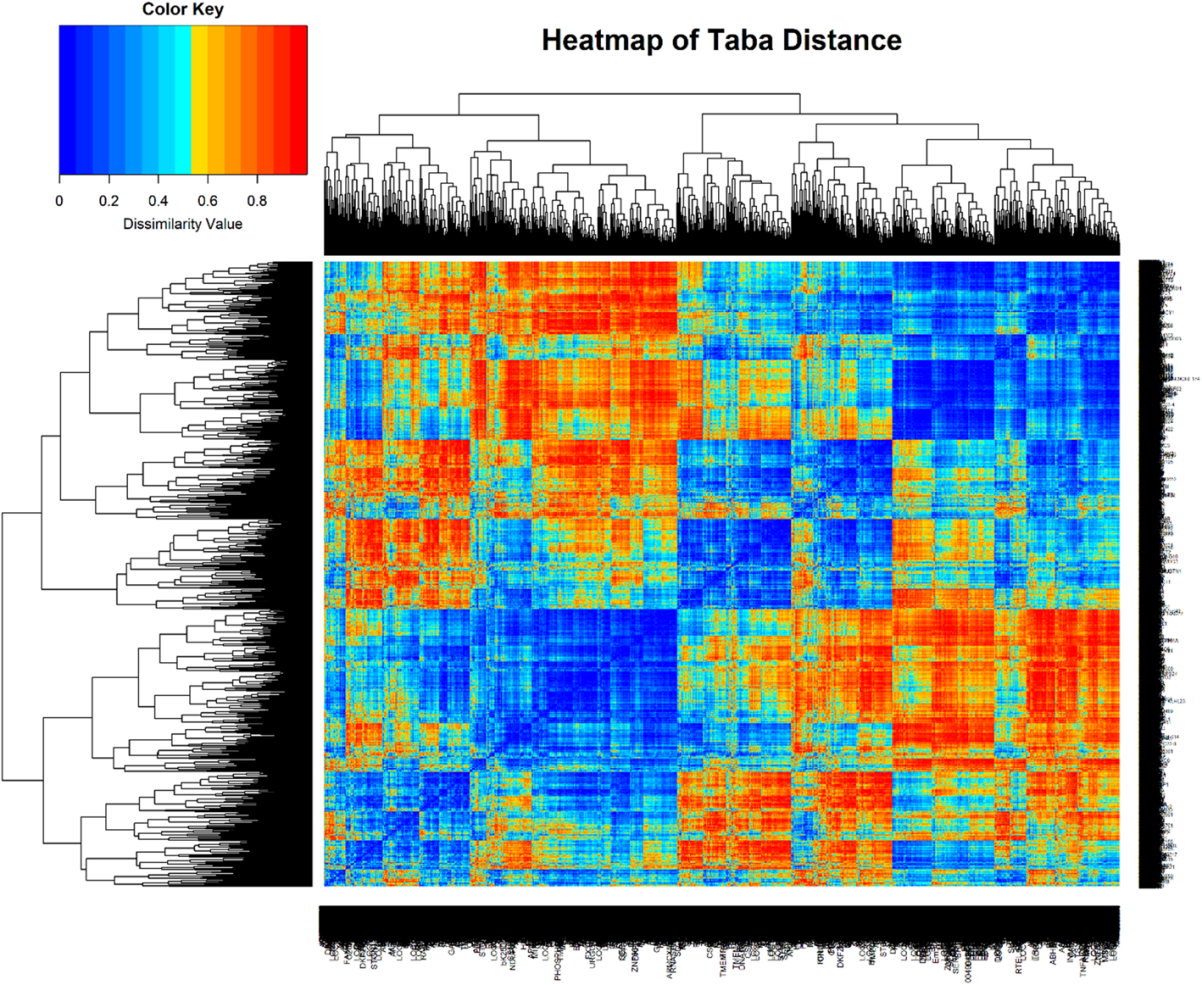
Heatmap of all 13,909 genes generated using RStudio software version 1.3.1073

A RNA transcriptome-based dataset of human gene expressions recorded in patients with Williams Syndrome (WS) was obtained from the National Center for Biotechnology Information (NCBI) [48]. Expression levels of genes that appeared in the dataset more than once were combined by averaging expression levels. Any genes with missing values were removed, resulting in a total sample size of 13,909 genes. Tissues were sampled from those with and without WS, each containing three replicated expressions. Silhouette and Elbow graphs were used to determine the optimal number of clusters. Figure 7 shows a visual snapshot of our gene expression data. It is a hierarchical clustering dendrogram for all genes using Taba distance.

After careful examination of data and checking the validity of assumptions, a one-sided t-test was conducted for each of the 13,909 individual genes to determine differences between control and WS groups. Statistical analysis of the data indicated only 43 genes had a *P *value less than 0.005 when control and WS groups were compared. The most statistically significant reduction in gene expression levels was associated with transducin beta-like 2 (TBL2) gene (*P *value = 6.37E−05). This gene encodes a member of the beta-transducin protein family known to be involved in regulatory functions. This gene is deleted in WS. The 2^nd^ most significant reduction in expression level of the WS group, when compared to control, was observed with the Bromodomain Adjacent to Zinc finger domain, 1B (BAZ1B) gene. BAZ1B plays an important role in neurodevelopment and implicate its haploinsufficiency as a likely contributor to the neurological phenotypes in WS [49]. Eukaryotic translation Initiation Factor 4H (EIF4H) encodes one of the translation initiation factors, which functions to stimulate the initiation of protein synthesis at the level of mRNA utilization. This gene is deleted in WS [50]. The top most significant genes belong to Chromosome 7.

We examined our entire dataset for the presence of outliers and found no significant outliers present. In order to demonstrate the extent of protection of all correlation measures considered in this article against outliers, we selected a random sample of one hundred genes from the set of 13,909, clustered them into two groups using each of the eight correlation estimators, and recorded the genes in each of the two clusters. One of the two groups were randomly selected and 10% of its genes were contaminated. For each selected gene, which consists of six replicates, one replicate was selected at random and contaminated. The contaminated replicate is equal to the value of the uncontaminated replicate plus ten times the standard deviation of the chosen gene prior to contamination. For each correlation measure, genes within both groups were reexamined and compared pre- and post-contamination. T, TW, TWR, and S had a perfect performance and had the same gene clustering results for pre- and post-contamination. Pearson had the worst performance, misplacing three genes. MCD, M, and Q misplaced only one gene when comparing pre- and post-clustered groups.

**Fig. 8:**
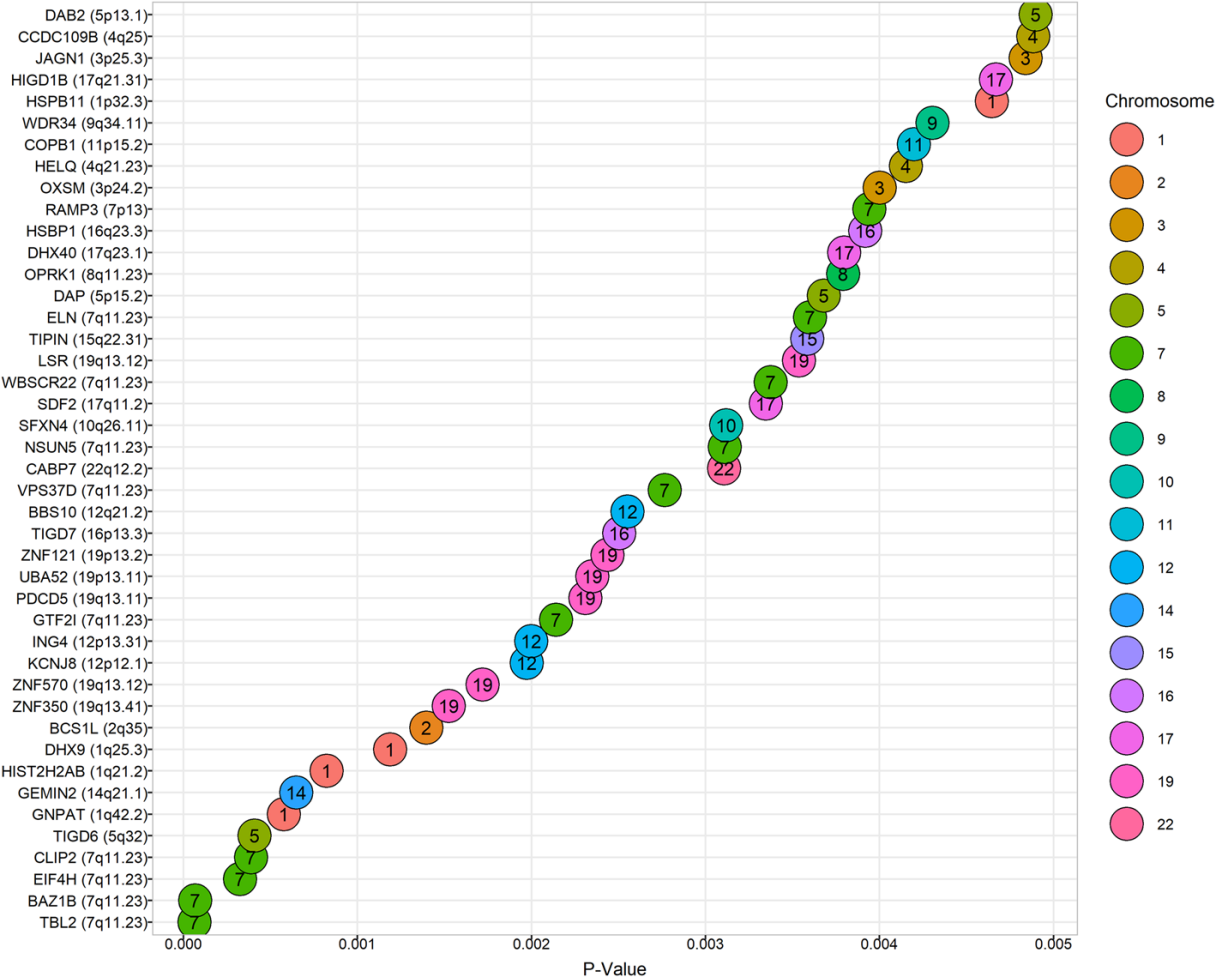
Ordered Forest Plot of 43 Genes (*P *value < 0.005) generated using RStudio software version 1.3.1073

Figure 8 shows the ordered forest plot of the top 43 genes having *P *values less than 0.005. The horizontal axis represents *P *values and the vertical axis represents genes. The numbers in the forest plot circles represent the chromosome each respective gene belongs to.

**Fig. 9:**
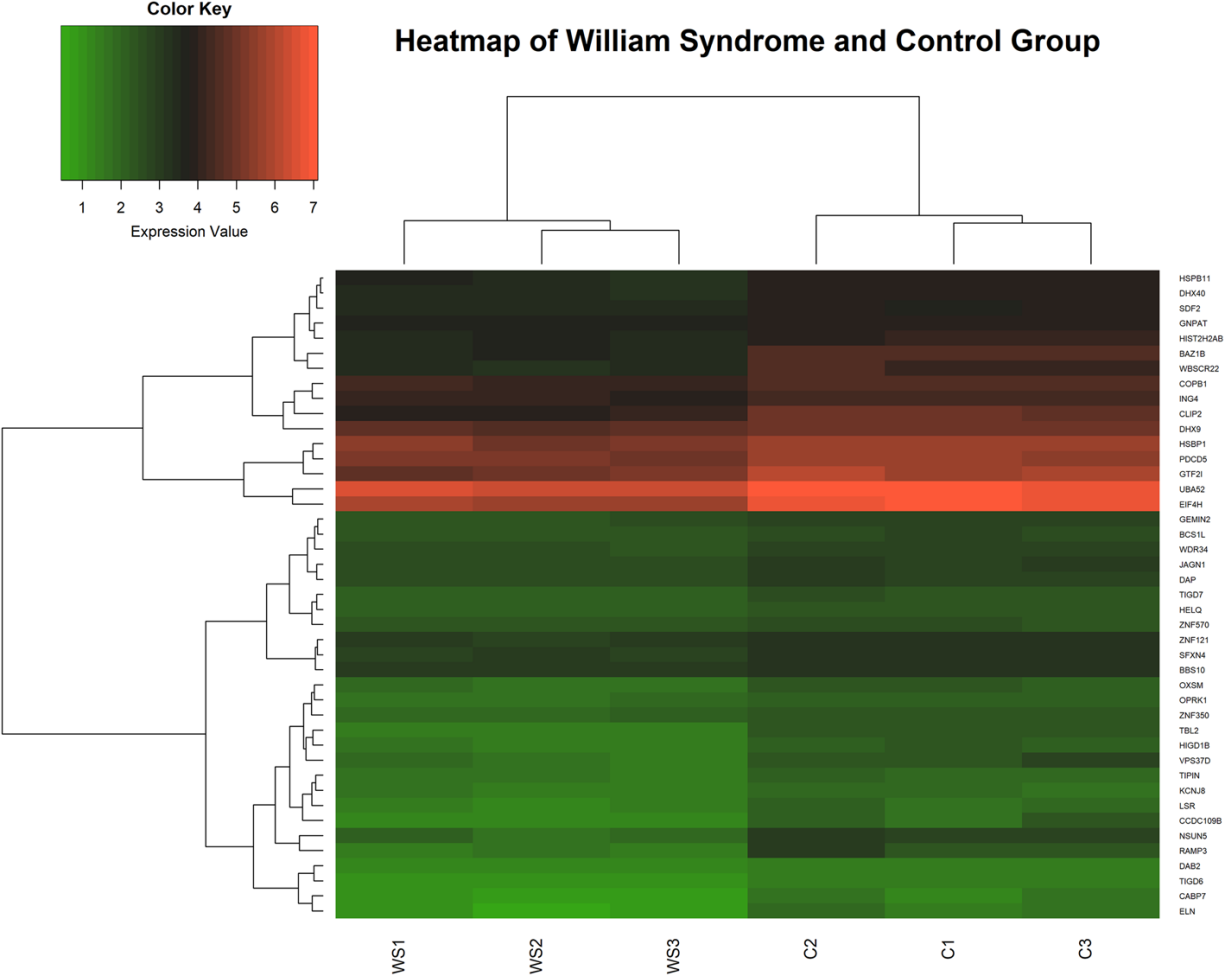
Clustered Heatmap of Genes with *P *values < 0.005 generated using RStudio software version 1.3.1073

Figure 9 illustrates the clustered heatmap using Taba dissimilarity measure for all genes having *P *values less than 0.005. The hierarchical clustering of samples clearly indicates two groups: the WS and the control. WS samples naturally clustered together and the same was observed for the control samples. The hierarchical clustering of the 43 genes shows at least two clusters. WS group has less intensity in colors when compared to the control group, indicating a significant difference in their expression levels between the two groups. In the cell consisting of WS samples and genes HSPB11, DHX40, SDF2, GNPAT, HIST2H2AB, BAZ1B, and WBSCR22, the most similar pair of genes were HSPB11 and DHX40, which belong to gene class B2 and B3 respectively. As far as we know there is no publication linking these two genes to WS. The next closest gene to these two genes was SDF2. GNPAT and HIST2H2AB were very close in their expression level and BAZ1B and WBSCR22 showed similar expression levels. The next block of genes in the WS category were COPB1, ING4, CLIP2, DHX9, HSBP1, PDCD5, GTF2I, UBA52, and EIF4H. COPB1, ING4, CLIP2, DHX9 and their expression levels showed similar patterns. HSBP1, PDCD5, and GTF2I had similar expression levels. The pair UBA52 and EIF4H were also expressed similarly. The remaining genes had a lower gene expression level when compared with the abovementioned genes.

## Taba R package

A statistical R package for calculating proposed robust measures is available (https://cran.r-project.org/web/packages/Taba/index.html). This package contains functions that evaluate correlations and their corresponding *P *values; partial and semi partial correlations; distance (dissimilarity) and *P *value matrices; as well as estimating generalized partial correlations. For partial, semi-partial, and generalized partial correlations, users will be able to specify the choice of link models such as linear, logistic, and Poisson for each outcome variable. The generalized partial correlation between two variables is similar to partial correlation, but will give the users the opportunity to control for different sets of confounding variables. If the two sets of confounding variables are identical, then the generalized partial correlation will reduce to a partial correlation.

## Conclusions

Robustness is a unique quality that not all frequently used measures have. Our work tackles an important issue in the usage of correlation coefficients either directly or indirectly as part of other various disciplines. Although MCD and P typically performed well under normal conditions, not all data follows a Normal distribution, however a majority of gene expression data is not normal [51]. When dealing with small samples from the bivariate Weibull or bivariate Log-Normal distributions the proposed methods are able to more accurately measure association between groups. By using an appropriate robust measure of correlation, one can improve the accuracy of the results and will enable researchers to better understand the true associations between variables in their models. It is imperative that a robust measure of correlation is used to reduce the severe impact of outliers. Thus, we recommend TabWil and Taba correlation for measuring linear association, and TabWil rank correlation for monotonic association because they are safeguards against the presence of outliers or influential observations.

Overall, MCD performed well based on bias and RMSE when the underlying distribution was bivariate Normal, but TabWil, TabWil rank, Taba, Spearman, and Pearson correlations were in close proximity. When the distribution was bivariate Log-Normal or bivariate Weibull, TabWil rank performed best in terms of bias but Taba performed best with respect to RMSE. Simulation results indicate that the proposed methods are highly robust and capable of determining the dissimilarity in large genomic datasets with thousands of genes, and hundreds of tissues.

Taba robust measure of distance was used to cluster genes using WS gene expression data. When comparing WS with the control group, TBL2 had the most significant reduction in its expression level. The gene TBL2, a possible regulator of the endoplasmic reticulum-resident kinase pathway expressed in a variety of organs such as the heart, skeletal muscle, and several endocrine tissues, can negatively affect nutrient conditions when deleted or under stress [52, 53]. This protein is often deleted in those diagnosed with WS [54, 55]. Other highly correlated genes such as BAZ1B, EIF4H, and CLIP2 are shown to be linked to conditions having similar effects [49, 56, 57].

TBL2 is associated with the eukaryotic 60S ribosomal subunit. This association was endoplasmic reticulum (ER) stress independent, but the TBL2-PERK (PKR-like ER-resident kinase) interaction occurred upon ER stress. This may help in understanding how TBL2 plays a role in the expression of proteins under ER stress [58]. Under ER stress, TBL2 partakes in Activating Transcription Factor 4 (ATF4) translation through its association with mRNA [59]. Furthermore, the deletion of TBL2, along with ER stress or poor nutrient conditions, can lead to impaired ATF4 induction. Thus, TBL2 serves as a potential regulator of the PERK pathway [52]. Due to the fact that haploinsufficiency has been shown for other Beta-Transducin repeat (WD-repeat) containing proteins, hemizygosity of TBL2 may have an impact on some aspects of the WS phenotype [60]. TBL2 has also been known to be highly associated with neurological syndromes [61]. Results suggest that TBL2 along with the 42 most significant genes identified in this study may serve as a diagnostic factor for WS. Future work includes investigating the robustness of the proposed methods in medical imaging and image recognition.

## Supplementary Information

**Additional file 1.** Simulation results produced using RStudio version 1.3.1073.

## Data Availability

Data can be found on the National Center for Biotechnology Information website (https://www.ncbi.nlm.nih.gov/geo/query/acc.cgi?acc=GSE128840). The Taba R Package is available on https://cran.r-project.org/web/packages/Taba/index.html.

## Abbreviations

P: Pearson correlation
S: Spearman correlation
Q: Quadrant correlation
M: Median correlation
MCD: Minimum covariance determinant correlation
T: Taba correlation
TW: TabWil correlation
TWR: TabWil rank correlation
WS: Williams syndrome
RMSE: Root mean square error
TBL2: Transducin Beta like 2
DNA: Deoxyribonucleic acid
Sech: Hyperbolic secant
Tanh: Hyperbolic tangent
ArcTanh: Inverse hyperbolic tangent
NCBI: National Center for Biotechnology Information
RNA: Ribonucleic acid
mRNA: Messenger ribonucleic acid
BAZ1B: Bromodomain Adjacent to Zinc finger domain, 1B
EIF4H: Eukaryotic translation Initiation Factor 4H
HSPB11: Heat Shock Protein Family B (Small) Member 11
DHX40: DEAH-Box Helicase 40
SDF2: Stromal Cell Derived Factor 2
GNPAT: Glyceronephosphate O-Acyltransferase
HIST2H2AB: H2A Clustered Histone 21
BAZ1B: Bromodomain adjacent to zinc finger domain, 1B
WBSCR22: Williams Beuren Syndrome Chromosome Region 22
COPB1: COPI Coat Complex Subunit Beta 1
ING4: Inhibitor of Growth Family Member 4
CLIP2: CAP-Gly Domain Containing Linker Protein 2
DHX9: DExH-Box Helicase 9
HSBP1: Heat Shock Factor Binding Protein 1
PDCD5: Programmed Cell Death 5
GTF2I: General Transcription Factor IIi
UBA52: Ubiquitin Carboxyl Extension Protein 52
EIF4H: Eukaryotic Translation Initiation Factor 4H
ING4: Inhibitor of Growth Family Member 4
CLIP2: CAP-Gly Domain Containing Linker Protein 2
DHX9: DExH-Box Helicase 9
ER: Endoplasmic Reticulum
PERK: PKR-like ER-resident kinase
ATF4: Activating Transcription Factor 4
WD-repeat: Beta-Transducin repeat
