# Correction to: An introduction to new robust linear and monotonic correlation coefficients

**DOI:** 10.1186/s12859-021-04244-y

**Published:** 2021-06-15

**Authors:** Mohammad Tabatabai, Stephanie Bailey, Zoran Bursac, Habib Tabatabai, Derek Wilus, Karan P. Singh

**Affiliations:** 1 Meharry Medical College, Nashville, TN 37208 USA; 2 Department of Biostatistics, Florida International University, Miami, FL 33199 USA; 3 Department of Civil and Environmental Engineering, University of Wisconsin Milwaukee, Milwaukee, WI 53211 USA; 4 Department of Epidemiology and Biostatistics, University of Texas Health Sciences Center at Tyler, Tyler, TX 75708 USA

## Correction to: BMC Bioinformatics (2021) 22:170 10.1186/s12859-021-04098-4

Following the publication of the original article [1], the authors identified an error in the equation. The correct equation is given below.

The incorrect equation:

$$t_{{(n - 2)}} = r_{{(\cdot)}} \sqrt {\frac{{n - 2}}{{1 - 0.5r_{{(\cdot)}}^{2} }}}$$

The correct equation:

$$t_{{(n - 2)}} = r_{{(\cdot)}} \sqrt {\frac{{n - 2}}{{1 - r_{{(\cdot)}}^{2} }}}$$

The original article [1] has been corrected.
