# Supplementary material for: An introduction to new robust linear and monotonic correlation coefficients
Source: BMC Bioinformatics. 2021 Mar 31;22:170. doi: 10.1186/s12859-021-04098-4 (PMC8011137; doi:10.1186/s12859-021-04098-4)
Supplement: Supplementary file 1 — Additional file 1. Simulation results produced using RStudio version 1.3.1073. [file 12859_2021_4098_MOESM1_ESM.pdf]

	n = 20		ρ = 0		Contamination = 0%			
	**Q**	**MCD**	**P**	**T**	**TW**	**M**	**S**	**TWR**
**Normal Bias**	0.000426	0.000903	0.001281	0.001656	0.001751	0.001952	0.002	0.003947
	**T**	**S**	**P**	**TW**	**M**	**Q**	**TWR**	**MCD**
**Normal RMSE**	0.227379	0.229756	0.231291	0.317596	0.319804	0.335725	0.376185	0.417003
	**MCD**	**P**	**S**	**Q**	**TWR**	**T**	**M**	**TW**
**Log-Normal Bias**	0.000691	0.005611	0.005893	0.006941	0.007422	0.007661	0.027051	0.0277
	**T**	**P**	**S**	**TW**	**M**	**Q**	**MCD**	**TWR**
**Log-Normal RMSE**	0.225807	0.229528	0.231	0.320412	0.322029	0.336217	0.375929	0.379071
	**TWR**	**S**	**P**	**Q**	**MCD**	**T**	**M**	**TW**
**Weibull Bias**	0.00014	0.001526	0.001562	0.00336	0.009674	0.01021	0.028931	0.031938
	**T**	**P**	**S**	**M**	**TW**	**Q**	**TWR**	**MCD**
**Weibull RMSE**	0.226762	0.230479	0.231527	0.330049	0.331092	0.341368	0.382626	0.405567
	n = 20		ρ = 0		Contamination = 5%			
	**Q**	**S**	**T**	**MCD**	**M**	**P**	**TW**	**TWR**
**Normal Bias**	0.000098	0.001075	0.001643	0.002124	0.002425	0.00304	0.004562	0.005733
	**T**	**P**	**S**	**M**	**TW**	**Q**	**TWR**	**MCD**
**Normal RMSE**	0.225102	0.226722	0.227064	0.316529	0.319198	0.33605	0.376968	0.388338
	**TWR**	**P**	**Q**	**MCD**	**S**	**T**	**M**	**TW**
**Log-Normal Bias**	0.000459	0.000535	0.000739	0.00089	0.001353	0.010845	0.035019	0.036353
	**T**	**P**	**S**	**TW**	**M**	**Q**	**MCD**	**TWR**
**Log-Normal RMSE**	0.224927	0.229663	0.23047	0.318081	0.318106	0.342892	0.363542	0.378796
	**P**	**S**	**Q**	**TWR**	**MCD**	**T**	**M**	**TW**
**Weibull Bias**	0.001579	0.001924	0.003518	0.003834	0.009177	0.014159	0.038779	0.040587
	**T**	**P**	**S**	**M**	**TW**	**Q**	**MCD**	**TWR**
**Weibull RMSE**	0.226888	0.229413	0.231237	0.326455	0.326795	0.339136	0.37523	0.379864
	n = 20		ρ = 0		Contamination = 10%			
	**P**	**S**	**Q**	**T**	**MCD**	**TWR**	**M**	**TW**
**Normal Bias**	0.000447	0.000705	0.001777	0.003168	0.003224	0.004813	0.026311	0.027256
	**T**	**S**	**P**	**TW**	**M**	**Q**	**MCD**	**TWR**
**Normal RMSE**	0.223709	0.228011	0.228289	0.312015	0.31392	0.34049	0.358443	0.376348
	**P**	**Q**	**MCD**	**S**	**TWR**	**T**	**M**	**TW**
**Log-Normal Bias**	0.002285	0.002359	0.003517	0.004881	0.005757	0.010796	0.046777	0.048332
	**T**	**S**	**P**	**M**	**TW**	**Q**	**MCD**	**TWR**
**Log-Normal RMSE**	0.221369	0.227182	0.22771	0.317356	0.31764	0.331474	0.34995	0.374785
	**P**	**MCD**	**S**	**TWR**	**Q**	**T**	**M**	**TW**
**Weibull Bias**	0.000045	0.001759	0.003672	0.005017	0.005598	0.015891	0.048592	0.048619
	**T**	**S**	**P**	**TW**	**M**	**Q**	**MCD**	**TWR**

**Weibull RMSE**	0.225933	0.229279	0.233476	0.321003	0.322949	0.339474	0.361928	0.379457
		**n = 20**		**ρ = 0.2**		**Contamination = 0%**		
	**P**	**T**	**S**	**MCD**	**Q**	**TWR**	**TW**	**M**
**Normal Bias**	0.002701	0.01208	0.015815	0.016159	0.016776	0.030045	0.033569	0.035787
	**T**	**P**	**S**	**TW**	**M**	**Q**	**TWR**	**MCD**
**Normal RMSE**	0.220624	0.222094	0.224068	0.31321	0.315755	0.329358	0.3595	0.406444
	**Q**	**S**	**TW**	**T**	**M**	**TWR**	**P**	**MCD**
**Log-Normal Bias**	0.018959	0.019489	0.021861	0.022344	0.024705	0.026371	0.026511	0.041874
	**T**	**S**	**P**	**TW**	**M**	**Q**	**TWR**	**MCD**
**Log-Normal RMSE**	0.222593	0.225819	0.234217	0.316666	0.317477	0.332545	0.365032	0.374195
	**TW**	**M**	**T**	**P**	**TWR**	**S**	**Q**	**MCD**
**Weibull Bias**	0.006769	0.012172	0.01466	0.018363	0.018616	0.021571	0.022653	0.026679
	**T**	**S**	**P**	**TW**	**M**	**Q**	**TWR**	**MCD**
**Weibull RMSE**	0.218519	0.221132	0.226287	0.314451	0.316087	0.329387	0.359045	0.393894
		**n = 20**		**ρ = 0.2**		**Contamination = 5%**		
	**TWR**	**MCD**	**TW**	**Q**	**M**	**T**	**S**	**P**
**Normal Bias**	0.005538	0.024182	0.038189	0.041409	0.043067	0.053026	0.054325	0.114695
	**T**	**S**	**P**	**TW**	**M**	**Q**	**TWR**	**MCD**
**Normal RMSE**	0.229785	0.23192	0.257081	0.311112	0.312193	0.334102	0.36751	0.378988
	**TW**	**M**	**TWR**	**Q**	**MCD**	**T**	**S**	**P**
**Log-Normal Bias**	0.007129	0.008384	0.016558	0.031189	0.036346	0.037806	0.048734	0.119567
	**T**	**S**	**P**	**TW**	**M**	**Q**	**MCD**	**TWR**
**Log-Normal RMSE**	0.221946	0.228364	0.26036	0.30216	0.302397	0.33419	0.353771	0.364412
	**M**	**TW**	**MCD**	**TWR**	**Q**	**T**	**S**	**P**
**Weibull Bias**	0.009674	0.011931	0.016239	0.018714	0.023898	0.035408	0.046938	0.114133
	**T**	**S**	**P**	**TW**	**M**	**Q**	**MCD**	**TWR**
**Weibull RMSE**	0.226218	0.233089	0.255501	0.31175	0.312099	0.333611	0.365884	0.367842
		**n = 20**		**ρ = 0.2**		**Contamination = 10%**		
	**TWR**	**TW**	**M**	**MCD**	**Q**	**S**	**T**	**P**
**Normal Bias**	0.003848	0.005537	0.011701	0.017112	0.043647	0.073944	0.079794	0.139028
	**S**	**T**	**P**	**TW**	**M**	**Q**	**MCD**	**TWR**
**Normal RMSE**	0.241909	0.24418	0.268476	0.304811	0.307723	0.337674	0.356978	0.36564
	**M**	**TW**	**TWR**	**MCD**	**Q**	**T**	**S**	**P**
**Log-Normal Bias**	0.000974	0.004976	0.018567	0.044843	0.050452	0.067459	0.074909	0.140844
	**T**	**S**	**P**	**M**	**TW**	**Q**	**MCD**	**TWR**
**Log-Normal RMSE**	0.234435	0.243221	0.273338	0.305744	0.306272	0.340444	0.351281	0.369688
	**M**	**TW**	**TWR**	**MCD**	**Q**	**T**	**S**	**P**

**Weibull Bias**	0.009369	0.012225	0.012326	0.027846	0.054978	0.062827	0.075993	0.145989
	**T**	**S**	**P**	**M**	**TW**	**Q**	**MCD**	**TWR**
**Weibull RMSE**	0.229699	0.23987	0.268375	0.305596	0.30627	0.338728	0.355156	0.367439
	**n = 20**			**ρ = 0.5**		**Contamination = 0%**		
	**P**	**T**	**MCD**	**S**	**Q**	**TWR**	**TW**	**M**
**Normal Bias**	0.013016	0.032894	0.03514	0.041728	0.043529	0.053405	0.079364	0.086135
	**P**	**T**	**S**	**TWR**	**TW**	**Q**	**M**	**MCD**
**Normal RMSE**	0.175614	0.183351	0.18964	0.28023	0.285532	0.289557	0.2898	0.347388
	**S**	**Q**	**P**	**T**	**TWR**	**MCD**	**TW**	**M**
**Log-Normal Bias**	0.034777	0.035023	0.048232	0.059291	0.061758	0.072377	0.072848	0.079612
	**S**	**T**	**P**	**TWR**	**TW**	**M**	**Q**	**MCD**
**Log-Normal RMSE**	0.19132	0.197102	0.209241	0.277082	0.280979	0.284099	0.290261	0.333473
	**P**	**T**	**S**	**Q**	**TWR**	**MCD**	**TW**	**M**
**Weibull Bias**	0.027755	0.042785	0.043256	0.04554	0.051821	0.05205	0.053307	0.062409
	**T**	**S**	**P**	**TWR**	**TW**	**M**	**Q**	**MCD**
**Weibull RMSE**	0.191693	0.193847	0.196327	0.284734	0.285417	0.289192	0.292921	0.351093
	**n = 20**			**ρ = 0.5**		**Contamination = 5%**		
	**TWR**	**MCD**	**TW**	**M**	**Q**	**T**	**S**	**P**
**Normal Bias**	0.010202	0.036298	0.074543	0.083421	0.086571	0.118582	0.126749	0.28675
	**T**	**S**	**TW**	**M**	**TWR**	**Q**	**MCD**	**P**
**Normal RMSE**	0.231684	0.239919	0.278647	0.285404	0.294864	0.312271	0.322081	0.355167
	**TWR**	**TW**	**M**	**Q**	**MCD**	**T**	**S**	**P**
**Log-Normal Bias**	0.018391	0.05845	0.064635	0.074929	0.076547	0.114918	0.122978	0.300193
	**T**	**S**	**TW**	**M**	**TWR**	**Q**	**MCD**	**P**
**Log-Normal RMSE**	0.227242	0.236124	0.269177	0.272003	0.291838	0.303379	0.317232	0.37066
	**TWR**	**TW**	**M**	**MCD**	**Q**	**T**	**S**	**P**
**Weibull Bias**	0.007967	0.043951	0.04983	0.052925	0.091071	0.114902	0.129286	0.292833
	**T**	**S**	**TW**	**M**	**TWR**	**Q**	**MCD**	**P**
**Weibull RMSE**	0.23001	0.242275	0.279806	0.282117	0.298445	0.317183	0.325571	0.362999
	**n = 20**			**ρ = 0.5**		**Contamination = 10%**		
	**MCD**	**TWR**	**TW**	**M**	**Q**	**S**	**T**	**P**
**Normal Bias**	0.034191	0.039936	0.061824	0.0699	0.129125	0.194454	0.207433	0.359428
	**TW**	**M**	**S**	**T**	**MCD**	**TWR**	**Q**	**P**
**Normal RMSE**	0.272597	0.276518	0.287655	0.298651	0.299356	0.313363	0.330867	0.421202
	**TWR**	**TW**	**M**	**MCD**	**Q**	**T**	**S**	**P**
**Log-Normal Bias**	0.035634	0.056127	0.061971	0.085364	0.128805	0.180252	0.194882	0.371719
	**TW**	**M**	**T**	**S**	**TWR**	**MCD**	**Q**	**P**

**Log-Normal RMSE**	0.267275	0.268412	0.271217	0.287871	0.308936	0.322924	0.32829	0.430811
	**TW**	**M**	**TWR**	**MCD**	**Q**	**T**	**S**	**P**
**Weibull Bias**	0.026911	0.032469	0.03577	0.049654	0.126591	0.181439	0.188287	0.355725
	**TW**	**M**	**T**	**S**	**MCD**	**TWR**	**Q**	**P**
**Weibull RMSE**	0.263908	0.266499	0.27673	0.2851	0.309471	0.312994	0.331383	0.419329
	**n = 20**			**ρ = 0.7**		**Contamination = 0%**		
	**P**	**T**	**MCD**	**S**	**Q**	**TWR**	**TW**	**M**
**Normal Bias**	0.008234	0.032151	0.035635	0.043167	0.045231	0.054695	0.086323	0.097516
	**P**	**T**	**S**	**TWR**	**Q**	**TW**	**M**	**MCD**
**Normal RMSE**	0.122329	0.135101	0.145064	0.187703	0.23184	0.234646	0.242574	0.267288
	**S**	**Q**	**P**	**TWR**	**T**	**MCD**	**TW**	**M**
**Log-Normal Bias**	0.045087	0.04944	0.053096	0.054575	0.075699	0.085585	0.099574	0.106792
	**S**	**P**	**T**	**TWR**	**Q**	**TW**	**M**	**MCD**
**Log-Normal RMSE**	0.147104	0.160526	0.164502	0.188515	0.236459	0.243887	0.250044	0.270026
	**P**	**S**	**T**	**Q**	**TWR**	**MCD**	**TW**	**M**
**Weibull Bias**	0.024216	0.045162	0.046362	0.047343	0.052974	0.054209	0.062811	0.073053
	**P**	**T**	**S**	**TWR**	**TW**	**M**	**Q**	**MCD**
**Weibull RMSE**	0.141113	0.145904	0.147703	0.186732	0.226787	0.234147	0.236678	0.271181
	**n = 20**			**ρ = 0.7**		**Contamination = 5%**		
	**TWR**	**MCD**	**TW**	**M**	**Q**	**T**	**S**	**P**
**Normal Bias**	0.012437	0.037431	0.087831	0.096951	0.106056	0.161274	0.169964	0.397992
	**TWR**	**TW**	**T**	**M**	**S**	**MCD**	**Q**	**P**
**Normal RMSE**	0.202337	0.232064	0.235715	0.237798	0.242819	0.244238	0.268703	0.444264
	**TWR**	**MCD**	**TW**	**M**	**Q**	**T**	**S**	**P**
**Log-Normal Bias**	0.016863	0.076587	0.084435	0.093472	0.103733	0.158505	0.168402	0.41917
	**TWR**	**TW**	**T**	**M**	**S**	**MCD**	**Q**	**P**
**Log-Normal RMSE**	0.197693	0.224016	0.226197	0.232218	0.239607	0.251016	0.259877	0.462646
	**TWR**	**MCD**	**TW**	**M**	**Q**	**T**	**S**	**P**
**Weibull Bias**	0.012284	0.047681	0.054502	0.06405	0.109441	0.151656	0.169213	0.403815
	**TWR**	**TW**	**M**	**T**	**S**	**MCD**	**Q**	**P**
**Weibull RMSE**	0.203562	0.214456	0.221669	0.227217	0.243724	0.248953	0.273081	0.449836
	**n = 20**			**ρ = 0.7**		**Contamination = 10%**		
	**MCD**	**TWR**	**TW**	**M**	**Q**	**S**	**T**	**P**
**Normal Bias**	0.025625	0.0418	0.07639	0.086737	0.165138	0.262676	0.282959	0.499279
	**MCD**	**TW**	**M**	**TWR**	**Q**	**S**	**T**	**P**
**Normal RMSE**	0.221649	0.225152	0.233411	0.238521	0.309352	0.328481	0.348944	0.543583
	**TWR**	**TW**	**MCD**	**M**	**Q**	**T**	**S**	**P**

**Log-Normal Bias**	0.039548	0.081485	0.083857	0.089574	0.160983	0.245937	0.259983	0.507233
	**TW**	**M**	**TWR**	**MCD**	**Q**	**T**	**S**	**P**
**Log-Normal RMSE**	0.221823	0.2273	0.234958	0.25126	0.3084	0.310475	0.326689	0.552187
	**TWR**	**TW**	**MCD**	**M**	**Q**	**T**	**S**	**P**
**Weibull Bias**	0.048402	0.05319	0.053398	0.061309	0.172344	0.267469	0.269418	0.506396
	**TW**	**M**	**TWR**	**MCD**	**Q**	**T**	**S**	**P**
**Weibull RMSE**	0.212892	0.218488	0.243695	0.244083	0.315565	0.331208	0.334341	0.54959
		**n = 20**		**ρ = 0.9**		**Contamination = 0%**		
	**P**	**MCD**	**T**	**TWR**	**S**	**Q**	**TW**	**M**
**Normal Bias**	0.004992	0.017504	0.021389	0.028059	0.032919	0.039461	0.057793	0.067608
	**P**	**T**	**TWR**	**S**	**MCD**	**TW**	**Q**	**M**
**Normal RMSE**	0.049815	0.062986	0.066266	0.074425	0.123986	0.130492	0.136911	0.142868
	**TWR**	**S**	**Q**	**P**	**MCD**	**T**	**TW**	**M**
**Log-Normal Bias**	0.025941	0.035175	0.042709	0.045863	0.056462	0.063266	0.081437	0.087856
	**TWR**	**S**	**P**	**T**	**Q**	**MCD**	**TW**	**M**
**Log-Normal RMSE**	0.066031	0.077253	0.081899	0.099284	0.140456	0.140938	0.14786	0.156893
	**P**	**MCD**	**TWR**	**T**	**S**	**Q**	**TW**	**M**
**Weibull Bias**	0.011182	0.027276	0.027558	0.031314	0.034049	0.04409	0.045801	0.056617
	**P**	**TWR**	**T**	**S**	**TW**	**MCD**	**M**	**Q**
**Weibull RMSE**	0.057961	0.065658	0.072568	0.075336	0.121916	0.127698	0.134446	0.140816
		**n = 20**		**ρ = 0.9**		**Contamination = 5%**		
	**TWR**	**MCD**	**TW**	**M**	**Q**	**T**	**S**	**P**
**Normal Bias**	0.002313	0.015823	0.060101	0.069315	0.100253	0.189675	0.197191	0.508147
	**TWR**	**MCD**	**TW**	**M**	**Q**	**T**	**S**	**P**
**Normal RMSE**	0.083005	0.113474	0.1342	0.144616	0.188397	0.239776	0.241921	0.538823
	**TWR**	**MCD**	**TW**	**M**	**Q**	**T**	**S**	**P**
**Log-Normal Bias**	0.002189	0.054304	0.077347	0.084968	0.104213	0.179709	0.1987	0.526916
	**TWR**	**MCD**	**TW**	**M**	**Q**	**T**	**S**	**P**
**Log-Normal RMSE**	0.081438	0.128353	0.137473	0.148602	0.192501	0.218272	0.243554	0.554932
	**TWR**	**MCD**	**TW**	**M**	**Q**	**T**	**S**	**P**
**Weibull Bias**	0.000039	0.027016	0.048521	0.058434	0.106299	0.178225	0.202911	0.515706
	**TWR**	**MCD**	**TW**	**M**	**Q**	**T**	**S**	**P**
**Weibull RMSE**	0.083693	0.119674	0.122191	0.134789	0.194982	0.220921	0.24814	0.542931
		**n = 20**		**ρ = 0.9**		**Contamination = 10%**		
	**MCD**	**TWR**	**TW**	**M**	**Q**	**S**	**T**	**P**
**Normal Bias**	0.013945	0.035437	0.067096	0.076411	0.172736	0.326661	0.35543	0.637941
	**MCD**	**TWR**	**TW**	**M**	**Q**	**S**	**T**	**P**

**Normal RMSE**	0.10339	0.117591	0.137465	0.149922	0.255565	0.372703	0.406402	0.670657
	**TWR**	**MCD**	**TW**	**M**	**Q**	**T**	**S**	**P**
**Log-Normal Bias**	0.033534	0.052906	0.075404	0.080992	0.170991	0.304483	0.323664	0.64864
	**TWR**	**MCD**	**TW**	**M**	**Q**	**T**	**S**	**P**
**Log-Normal RMSE**	0.115363	0.121536	0.134742	0.139697	0.253382	0.347502	0.369794	0.679735
	**MCD**	**TWR**	**TW**	**M**	**Q**	**S**	**T**	**P**
**Weibull Bias**	0.022513	0.034128	0.048356	0.054748	0.170102	0.325164	0.325176	0.640051
	**MCD**	**TWR**	**TW**	**M**	**Q**	**S**	**T**	**P**
**Weibull RMSE**	0.10288	0.115213	0.118276	0.125794	0.253618	0.371002	0.37161	0.67111
	**n = 40 ρ = 0 Contamination = 0%**							
	**S**	**T**	**TWR**	**P**	**M**	**TW**	**MCD**	**Q**
**Normal Bias**	0.000408	0.000478	0.000861	0.001379	0.001478	0.002758	0.003312	0.003628
	**T**	**P**	**S**	**M**	**TW**	**Q**	**TWR**	**MCD**
**Normal RMSE**	0.159153	0.160501	0.160947	0.241919	0.242235	0.246438	0.286003	0.290465
	**S**	**P**	**Q**	**MCD**	**TWR**	**T**	**M**	**TW**
**Log-Normal Bias**	0.000763	0.000973	0.003323	0.003443	0.004976	0.014453	0.042042	0.04225
	**T**	**S**	**P**	**Q**	**TW**	**M**	**MCD**	**TWR**
**Log-Normal RMSE**	0.158442	0.160232	0.160411	0.242823	0.245359	0.245914	0.250116	0.284884
	**S**	**Q**	**P**	**TWR**	**MCD**	**T**	**M**	**TW**
**Weibull Bias**	0.002308	0.002525	0.002786	0.003761	0.007175	0.01541	0.036031	0.037461
	**T**	**S**	**P**	**Q**	**M**	**TW**	**MCD**	**TWR**
**Weibull RMSE**	0.159168	0.159812	0.161501	0.243454	0.247522	0.248162	0.270272	0.286696
	**n = 40 ρ = 0 Contamination = 5%**							
	**TWR**	**Q**	**S**	**MCD**	**T**	**P**	**M**	**TW**
**Normal Bias**	0.000105	0.000553	0.0013	0.001652	0.001855	0.002756	0.008445	0.008535
	**T**	**P**	**S**	**TW**	**M**	**Q**	**MCD**	**TWR**
**Normal RMSE**	0.157018	0.157739	0.15834	0.236297	0.236741	0.239511	0.260264	0.281819
	**TWR**	**Q**	**S**	**P**	**MCD**	**T**	**M**	**TW**
**Log-Normal Bias**	0.000241	0.000633	0.000929	0.001491	0.010103	0.011347	0.044242	0.046182
	**T**	**S**	**P**	**MCD**	**M**	**TW**	**Q**	**TWR**
**Log-Normal RMSE**	0.15721	0.159923	0.160323	0.235506	0.241436	0.241977	0.243974	0.281468
	**Q**	**TWR**	**P**	**S**	**MCD**	**T**	**TW**	**M**
**Weibull Bias**	0.000916	0.0016	0.001918	0.002039	0.008239	0.015393	0.042984	0.04352
	**T**	**S**	**P**	**Q**	**M**	**TW**	**MCD**	**TWR**
**Weibull RMSE**	0.155293	0.156411	0.159845	0.241016	0.242994	0.24415	0.248132	0.280784
	**n = 40 ρ = 0 Contamination = 10%**							
	**T**	**Q**	**S**	**P**	**TWR**	**MCD**	**M**	**TW**

**Normal Bias**	0.000062	0.000743	0.001363	0.003484	0.003771	0.007679	0.029773	0.030347
	**T**	**P**	**S**	**M**	**TW**	**MCD**	**Q**	**TWR**
**Normal RMSE**	0.157797	0.158604	0.158716	0.234027	0.234126	0.24138	0.246525	0.28588
	**TWR**	**S**	**Q**	**P**	**T**	**MCD**	**TW**	**M**
**Log-Normal Bias**	0.000144	0.001184	0.002276	0.002567	0.016064	0.018826	0.057273	0.057708
	**T**	**P**	**S**	**MCD**	**TW**	**M**	**Q**	**TWR**
**Log-Normal RMSE**	0.158779	0.159426	0.161365	0.232321	0.239967	0.240064	0.240514	0.285745
	**S**	**Q**	**P**	**TWR**	**MCD**	**T**	**M**	**TW**
**Weibull Bias**	0.004018	0.004136	0.005113	0.005163	0.011211	0.023291	0.060956	0.061221
	**T**	**P**	**S**	**MCD**	**Q**	**M**	**TW**	**TWR**
**Weibull RMSE**	0.160539	0.161339	0.163284	0.241885	0.245996	0.250681	0.251479	0.288748
		**n = 40**		**ρ = 0.2**		**Contamination = 0%**		
	**P**	**Q**	**MCD**	**T**	**S**	**TW**	**M**	**TWR**
**Normal Bias**	0.003549	0.007484	0.009	0.011403	0.014829	0.01966	0.02289	0.046843
	**T**	**P**	**S**	**TW**	**M**	**Q**	**TWR**	**MCD**
**Normal RMSE**	0.153696	0.15402	0.155325	0.233118	0.235544	0.235565	0.271203	0.27141
	**M**	**TW**	**Q**	**S**	**T**	**P**	**MCD**	**TWR**
**Log-Normal Bias**	0.003674	0.005515	0.006864	0.01059	0.013712	0.02467	0.04295	0.051238
	**T**	**S**	**P**	**M**	**TW**	**Q**	**MCD**	**TWR**
**Log-Normal RMSE**	0.152932	0.156274	0.167076	0.233507	0.23417	0.24039	0.253816	0.27465
	**T**	**Q**	**P**	**S**	**M**	**TW**	**MCD**	**TWR**
**Weibull Bias**	0.00632	0.007121	0.01266	0.012809	0.015307	0.018156	0.020679	0.047834
	**T**	**S**	**P**	**Q**	**TW**	**M**	**MCD**	**TWR**
**Weibull RMSE**	0.150758	0.152989	0.158148	0.233097	0.235233	0.236336	0.267099	0.268618
		**n = 40**		**ρ = 0.2**		**Contamination = 5%**		
	**MCD**	**TW**	**M**	**TWR**	**Q**	**T**	**S**	**P**
**Normal Bias**	0.008325	0.013029	0.016455	0.025127	0.028074	0.042269	0.045456	0.107227
	**T**	**S**	**P**	**TW**	**M**	**Q**	**MCD**	**TWR**
**Normal RMSE**	0.15985	0.161853	0.190123	0.23113	0.23142	0.240183	0.251059	0.270859
	**M**	**TW**	**TWR**	**Q**	**T**	**MCD**	**S**	**P**
**Log-Normal Bias**	0.009857	0.010711	0.023908	0.029419	0.041168	0.049219	0.05108	0.125679
	**T**	**S**	**P**	**TW**	**M**	**Q**	**MCD**	**TWR**
**Log-Normal RMSE**	0.157578	0.164246	0.202785	0.22667	0.227706	0.23643	0.238942	0.272194
	**M**	**TW**	**TWR**	**Q**	**MCD**	**T**	**S**	**P**
**Weibull Bias**	0.01789	0.020389	0.025556	0.026048	0.028541	0.033153	0.04622	0.116056
	**T**	**S**	**P**	**M**	**TW**	**Q**	**MCD**	**TWR**
**Weibull RMSE**	0.158286	0.165037	0.196908	0.239271	0.239521	0.242287	0.252305	0.27511

	n = 40			ρ = 0.2		Contamination = 10%		
	TW	M	TWR	MCD	Q	S	T	P
**Normal Bias**	0.000809	0.002789	0.003439	0.016791	0.050579	0.078956	0.081858	0.144178
	S	T	P	TW	M	MCD	Q	TWR
**Normal RMSE**	0.175841	0.176068	0.214282	0.227864	0.228008	0.235664	0.245374	0.273969
	TWR	M	TW	Q	MCD	T	S	P
**Log-Normal Bias**	0.001852	0.015393	0.018054	0.048015	0.054673	0.060175	0.074639	0.145602
	T	S	P	M	TW	MCD	Q	TWR
**Log-Normal RMSE**	0.166515	0.175016	0.217675	0.226447	0.22652	0.238347	0.241264	0.273295
	TWR	M	MCD	TW	Q	T	S	P
**Weibull Bias**	0.001563	0.033704	0.035223	0.035241	0.047944	0.060213	0.073288	0.140603
	T	S	P	TW	M	MCD	Q	TWR
**Weibull RMSE**	0.165984	0.175	0.21335	0.234615	0.234705	0.237294	0.244463	0.277013
	n = 40			ρ = 0.5		Contamination = 0%		
	P	MCD	T	Q	S	TW	M	TWR
**Normal Bias**	0.00625	0.021373	0.022284	0.023623	0.030385	0.043703	0.049185	0.087952
	P	T	S	TW	M	Q	TWR	MCD
**Normal RMSE**	0.121696	0.126452	0.131396	0.198189	0.201727	0.202417	0.209055	0.229002
	Q	S	TW	P	M	T	MCD	TWR
**Log-Normal Bias**	0.021268	0.030318	0.048169	0.050779	0.051201	0.053909	0.087369	0.087567
	S	T	P	TW	M	Q	TWR	MCD
**Log-Normal RMSE**	0.134536	0.1434	0.15483	0.202058	0.203571	0.20453	0.209767	0.234113
	TW	M	Q	P	T	S	MCD	TWR
**Weibull Bias**	0.009192	0.013416	0.019843	0.022722	0.029599	0.029641	0.045769	0.087634
	S	T	P	TW	M	Q	TWR	MCD
**Weibull RMSE**	0.13239	0.132582	0.137428	0.194947	0.196676	0.202808	0.209227	0.230102
	n = 40			ρ = 0.5		Contamination = 5%		
	MCD	TW	M	TWR	Q	T	S	P
**Normal Bias**	0.013581	0.036607	0.042893	0.049856	0.066181	0.106623	0.112974	0.275885
	T	S	TW	M	MCD	TWR	Q	P
**Normal RMSE**	0.173771	0.178804	0.193726	0.197973	0.203477	0.206862	0.220569	0.3113
	TW	M	TWR	Q	MCD	T	S	P
**Log-Normal Bias**	0.036965	0.039234	0.048235	0.063479	0.08483	0.104713	0.114799	0.298558
	T	S	TW	M	TWR	Q	MCD	P
**Log-Normal RMSE**	0.172009	0.182273	0.196444	0.196815	0.209399	0.218862	0.222489	0.334526
	TW	M	MCD	TWR	Q	T	S	P
**Weibull Bias**	0.001023	0.003074	0.043231	0.052339	0.064617	0.100151	0.114805	0.283489
	T	S	TW	M	TWR	MCD	Q	P

**Weibull RMSE**	0.167697	0.179146	0.187651	0.187677	0.203053	0.215105	0.217629	0.320044
		**n = 40**		**ρ = 0.5**		**Contamination = 10%**		
	**TWR**	**MCD**	**TW**	**M**	**Q**	**S**	**T**	**P**
**Normal Bias**	0.000675	0.016047	0.028139	0.035061	0.110761	0.179646	0.193122	0.347659
	**TW**	**M**	**MCD**	**TWR**	**S**	**Q**	**T**	**P**
**Normal RMSE**	0.18964	0.191299	0.192015	0.222061	0.233967	0.242866	0.245565	0.379763
	**TWR**	**TW**	**M**	**MCD**	**Q**	**T**	**S**	**P**
**Log-Normal Bias**	0.004909	0.026573	0.030271	0.086892	0.113369	0.165865	0.181984	0.363836
	**TW**	**M**	**T**	**TWR**	**MCD**	**S**	**Q**	**P**
**Log-Normal RMSE**	0.190661	0.191755	0.218512	0.220949	0.221741	0.234721	0.245574	0.394745
	**M**	**TWR**	**TW**	**MCD**	**Q**	**T**	**S**	**P**
**Weibull Bias**	0.00054	0.00181	0.002642	0.051153	0.111603	0.173072	0.181245	0.350252
	**TW**	**M**	**MCD**	**TWR**	**T**	**S**	**Q**	**P**
**Weibull RMSE**	0.187098	0.187721	0.210076	0.222458	0.22718	0.235115	0.246854	0.383288
		**n = 40**		**ρ = 0.7**		**Contamination = 0%**		
	**P**	**MCD**	**Q**	**T**	**S**	**TW**	**M**	**TWR**
**Normal Bias**	0.005831	0.013583	0.021783	0.023043	0.031894	0.042661	0.050607	0.08584
	**P**	**T**	**S**	**TWR**	**TW**	**M**	**MCD**	**Q**
**Normal RMSE**	0.084722	0.093228	0.100173	0.138242	0.150893	0.156729	0.157972	0.160611
	**Q**	**S**	**P**	**TW**	**T**	**M**	**TWR**	**MCD**
**Log-Normal Bias**	0.027728	0.033375	0.058187	0.062587	0.066138	0.067428	0.082356	0.086303
	**S**	**T**	**P**	**TWR**	**Q**	**TW**	**M**	**MCD**
**Log-Normal RMSE**	0.101715	0.12061	0.125025	0.139263	0.161217	0.161718	0.166351	0.187851
	**TW**	**P**	**M**	**Q**	**S**	**T**	**MCD**	**TWR**
**Weibull Bias**	0.014512	0.019866	0.020167	0.021407	0.030323	0.033092	0.040147	0.084135
	**P**	**S**	**T**	**TWR**	**TW**	**M**	**Q**	**MCD**
**Weibull RMSE**	0.100761	0.100938	0.101789	0.140116	0.143942	0.147432	0.159964	0.17445
		**n = 40**		**ρ = 0.7**		**Contamination = 5%**		
	**MCD**	**TWR**	**TW**	**M**	**Q**	**T**	**S**	**P**
**Normal Bias**	0.017411	0.04466	0.047112	0.053121	0.082761	0.147741	0.15441	0.386504
	**TWR**	**MCD**	**TW**	**M**	**Q**	**T**	**S**	**P**
**Normal RMSE**	0.137729	0.150094	0.154582	0.159109	0.190335	0.191244	0.196365	0.409175
	**TWR**	**TW**	**M**	**Q**	**MCD**	**T**	**S**	**P**
**Log-Normal Bias**	0.046179	0.0523	0.057084	0.080494	0.080882	0.140154	0.152157	0.411697
	**TWR**	**TW**	**M**	**MCD**	**T**	**Q**	**S**	**P**
**Log-Normal RMSE**	0.138972	0.152298	0.156205	0.176453	0.179204	0.19168	0.193283	0.43369
	**TW**	**M**	**MCD**	**TWR**	**Q**	**T**	**S**	**P**

**Weibull Bias**	0.01406	0.018805	0.04126	0.044189	0.0853	0.138792	0.157453	0.3973
	**TWR**	**TW**	**M**	**MCD**	**T**	**Q**	**S**	**P**
**Weibull RMSE**	0.136713	0.139217	0.142648	0.160318	0.181566	0.1917	0.198851	0.419856
		**n = 40**		**ρ = 0.7**		**Contamination = 10%**		
	**TWR**	**MCD**	**TW**	**M**	**Q**	**S**	**T**	**P**
**Normal Bias**	0.001728	0.011256	0.042385	0.049228	0.136696	0.246471	0.26904	0.48709
	**MCD**	**TW**	**TWR**	**M**	**Q**	**S**	**T**	**P**
**Normal RMSE**	0.133979	0.151668	0.152457	0.156588	0.229214	0.281623	0.304583	0.508734
	**TWR**	**TW**	**M**	**MCD**	**Q**	**T**	**S**	**P**
**Log-Normal Bias**	0.001153	0.044091	0.048387	0.081467	0.139718	0.226438	0.244994	0.504185
	**TW**	**M**	**TWR**	**MCD**	**Q**	**T**	**S**	**P**
**Log-Normal RMSE**	0.145729	0.148729	0.149999	0.173344	0.228288	0.260181	0.279489	0.525057
	**TWR**	**TW**	**M**	**MCD**	**Q**	**T**	**S**	**P**
**Weibull Bias**	0.002287	0.00739	0.012206	0.041123	0.140463	0.242239	0.246716	0.490737
	**TW**	**M**	**TWR**	**MCD**	**Q**	**T**	**S**	**P**
**Weibull RMSE**	0.137792	0.139359	0.153042	0.156292	0.231578	0.27719	0.281971	0.512029
		**n = 40**		**ρ = 0.9**		**Contamination = 0%**		
	**P**	**MCD**	**T**	**Q**	**S**	**TW**	**M**	**TWR**
**Normal Bias**	0.002949	0.008012	0.014628	0.02173	0.022298	0.028212	0.034402	0.038257
	**P**	**T**	**S**	**TWR**	**MCD**	**TW**	**M**	**Q**
**Normal RMSE**	0.032008	0.040367	0.047856	0.050564	0.068114	0.072229	0.079108	0.088557
	**Q**	**S**	**TWR**	**P**	**T**	**MCD**	**TW**	**M**
**Log-Normal Bias**	0.021511	0.021851	0.038297	0.050886	0.054567	0.055338	0.058263	0.061331
	**S**	**TWR**	**P**	**T**	**Q**	**MCD**	**TW**	**M**
**Log-Normal RMSE**	0.047345	0.050317	0.07114	0.073672	0.08851	0.093332	0.094936	0.099625
	**P**	**TW**	**MCD**	**M**	**Q**	**T**	**S**	**TWR**
**Weibull Bias**	0.007974	0.013572	0.018026	0.018969	0.020385	0.021198	0.021292	0.03911
	**P**	**T**	**S**	**TWR**	**TW**	**M**	**MCD**	**Q**
**Weibull RMSE**	0.039529	0.048076	0.048182	0.051677	0.064762	0.069466	0.074217	0.087393
		**n = 40**		**ρ = 0.9**		**Contamination = 5%**		
	**MCD**	**TWR**	**TW**	**M**	**Q**	**T**	**S**	**P**
**Normal Bias**	0.006521	0.019317	0.031178	0.03758	0.078672	0.178653	0.182894	0.494889
	**TWR**	**MCD**	**TW**	**M**	**Q**	**T**	**S**	**P**
**Normal RMSE**	0.048053	0.058705	0.073565	0.080944	0.13139	0.205488	0.206461	0.509341
	**TWR**	**TW**	**MCD**	**M**	**Q**	**T**	**S**	**P**
**Log-Normal Bias**	0.020105	0.05361	0.054283	0.058076	0.079043	0.157735	0.178963	0.516257
	**TWR**	**TW**	**MCD**	**M**	**Q**	**T**	**S**	**P**

**Log-Normal RMSE**	0.049947	0.091275	0.091547	0.096972	0.133659	0.178191	0.203039	0.530014
	**TW**	**MCD**	**TWR**	**M**	**Q**	**T**	**S**	**P**
**Weibull Bias**	0.015049	0.019351	0.019809	0.019814	0.07787	0.160431	0.182245	0.497292
	**TWR**	**TW**	**M**	**MCD**	**Q**	**T**	**S**	**P**
**Weibull RMSE**	0.04899	0.062232	0.067813	0.068821	0.131499	0.184779	0.206667	0.511229
	**n = 40**			**ρ = 0.9**		**Contamination = 10%**		
	**MCD**	**TWR**	**TW**	**M**	**Q**	**S**	**T**	**P**
**Normal Bias**	0.005285	0.007986	0.033154	0.037976	0.140121	0.30493	0.338713	0.622722
	**MCD**	**TWR**	**TW**	**M**	**Q**	**S**	**T**	**P**
**Normal RMSE**	0.051129	0.060045	0.075324	0.080918	0.186856	0.328099	0.364219	0.637948
	**TWR**	**TW**	**MCD**	**M**	**Q**	**T**	**S**	**P**
**Log-Normal Bias**	0.006928	0.051158	0.052253	0.05614	0.139738	0.279035	0.302182	0.639863
	**TWR**	**MCD**	**TW**	**M**	**Q**	**T**	**S**	**P**
**Log-Normal RMSE**	0.061549	0.086319	0.08718	0.092195	0.187474	0.301818	0.326004	0.654413
	**TWR**	**TW**	**MCD**	**M**	**Q**	**T**	**S**	**P**
**Weibull Bias**	0.008621	0.015347	0.019157	0.019539	0.144721	0.308862	0.309421	0.630007
	**TWR**	**TW**	**MCD**	**M**	**Q**	**S**	**T**	**P**
**Weibull RMSE**	0.060897	0.062164	0.065518	0.065771	0.193091	0.333453	0.333732	0.645656
	**n = 80**			**ρ = 0**		**Contamination = 0%**		
	**Q**	**TW**	**M**	**S**	**T**	**P**	**TWR**	**MCD**
**Normal Bias**	0.000128	0.000168	0.000236	0.000463	0.000752	0.000787	0.001439	0.001692
	**P**	**T**	**S**	**Q**	**TW**	**M**	**MCD**	**TWR**
**Normal RMSE**	0.111332	0.111467	0.112275	0.173235	0.17531	0.175967	0.190335	0.206265
	**S**	**Q**	**TWR**	**P**	**T**	**MCD**	**M**	**TW**
**Log-Normal Bias**	0.000096	0.000308	0.001518	0.001606	0.014533	0.022441	0.04423	0.045011
	**T**	**S**	**P**	**MCD**	**Q**	**M**	**TW**	**TWR**
**Log-Normal RMSE**	0.111875	0.113394	0.115334	0.17184	0.175526	0.182632	0.182805	0.210997
	**P**	**TWR**	**S**	**Q**	**T**	**MCD**	**M**	**TW**
**Weibull Bias**	0.002694	0.002768	0.002783	0.00294	0.011118	0.024066	0.04133	0.042089
	**T**	**P**	**S**	**Q**	**M**	**MCD**	**TW**	**TWR**
**Weibull RMSE**	0.11154	0.112836	0.11293	0.173582	0.186241	0.186279	0.186411	0.20792
	**n = 80**			**ρ = 0**		**Contamination = 5%**		
	**MCD**	**S**	**Q**	**T**	**P**	**TWR**	**M**	**TW**
**Normal Bias**	0.00013	0.001975	0.00201	0.002506	0.002562	0.003722	0.008488	0.008861
	**T**	**S**	**P**	**Q**	**MCD**	**TW**	**M**	**TWR**
**Normal RMSE**	0.110492	0.111079	0.113267	0.171128	0.171438	0.17311	0.173692	0.205973
	**S**	**Q**	**TWR**	**P**	**T**	**MCD**	**M**	**TW**

**Log-Normal Bias**	0.001149	0.001983	0.002028	0.00236	0.014624	0.021622	0.050016	0.050179
	**P**	**S**	**T**	**MCD**	**Q**	**TW**	**M**	**TWR**
**Log-Normal RMSE**	0.112897	0.113641	0.11403	0.164016	0.177009	0.181462	0.182016	0.211337
	**P**	**S**	**TWR**	**Q**	**T**	**MCD**	**M**	**TW**
**Weibull Bias**	0.000699	0.001644	0.00319	0.003222	0.012719	0.027043	0.045564	0.045641
	**P**	**T**	**S**	**MCD**	**Q**	**TW**	**M**	**TWR**
**Weibull RMSE**	0.112774	0.112901	0.113206	0.174248	0.176947	0.186138	0.18648	0.211531
		**n = 80**		**ρ = 0**		**Contamination = 10%**		
	**Q**	**S**	**TWR**	**MCD**	**P**	**T**	**M**	**TW**
**Normal Bias**	0.00045	0.001605	0.002075	0.002279	0.002724	0.003386	0.029936	0.030604
	**P**	**T**	**S**	**MCD**	**TW**	**M**	**Q**	**TWR**
**Normal RMSE**	0.112338	0.112701	0.11281	0.164941	0.173612	0.17435	0.174872	0.210205
	**P**	**S**	**TWR**	**Q**	**T**	**MCD**	**TW**	**M**
**Log-Normal Bias**	0.002274	0.003331	0.004335	0.005474	0.018571	0.022726	0.063179	0.063221
	**P**	**S**	**T**	**MCD**	**Q**	**M**	**TW**	**TWR**
**Log-Normal RMSE**	0.111043	0.11228	0.113105	0.158994	0.17658	0.18446	0.185178	0.209944
	**P**	**S**	**Q**	**TWR**	**T**	**MCD**	**TW**	**M**
**Weibull Bias**	0.000001	0.002611	0.002971	0.005353	0.016916	0.026821	0.058712	0.059727
	**T**	**S**	**P**	**MCD**	**Q**	**TW**	**M**	**TWR**
**Weibull RMSE**	0.112559	0.112925	0.1138	0.16224	0.17554	0.188058	0.188623	0.211464
		**n = 80**		**ρ = 0.2**		**Contamination = 0%**		
	**P**	**MCD**	**Q**	**T**	**S**	**TW**	**M**	**TWR**
**Normal Bias**	0.001937	0.004392	0.005458	0.008393	0.011582	0.013326	0.014996	0.058146
	**P**	**T**	**S**	**Q**	**TW**	**M**	**MCD**	**TWR**
**Normal RMSE**	0.108024	0.108635	0.109571	0.168205	0.173331	0.173637	0.190306	0.205256
	**Q**	**M**	**TW**	**S**	**T**	**P**	**TWR**	**MCD**
**Log-Normal Bias**	0.006729	0.009439	0.00994	0.012091	0.015398	0.027756	0.057082	0.058124
	**T**	**S**	**P**	**Q**	**M**	**TW**	**MCD**	**TWR**
**Log-Normal RMSE**	0.109828	0.110805	0.12427	0.169429	0.1699	0.170716	0.177325	0.204135
	**T**	**Q**	**S**	**P**	**M**	**MCD**	**TW**	**TWR**
**Weibull Bias**	0.001161	0.004341	0.008424	0.008848	0.033692	0.034203	0.034209	0.061102
	**T**	**S**	**P**	**Q**	**M**	**TW**	**MCD**	**TWR**
**Weibull RMSE**	0.109122	0.110169	0.11505	0.170811	0.176276	0.176536	0.190259	0.205137
		**n = 80**		**ρ = 0.2**		**Contamination = 5%**		
	**MCD**	**TW**	**M**	**Q**	**T**	**TWR**	**S**	**P**
**Normal Bias**	0.002608	0.004607	0.005853	0.0203	0.038932	0.039233	0.042175	0.105533
	**T**	**S**	**P**	**MCD**	**TW**	**M**	**Q**	**TWR**

**Normal RMSE**	0.115125	0.116568	0.152678	0.164674	0.169415	0.169576	0.170125	0.199397
	**M**	**TW**	**Q**	**T**	**TWR**	**S**	**MCD**	**P**
**Log-Normal Bias**	0.018418	0.019414	0.024618	0.032809	0.035235	0.044148	0.05685	0.122499
	**T**	**S**	**P**	**M**	**TW**	**MCD**	**Q**	**TWR**
**Log-Normal RMSE**	0.11476	0.121032	0.167131	0.169425	0.17041	0.172506	0.174046	0.20348
	**Q**	**T**	**TWR**	**TW**	**M**	**MCD**	**S**	**P**
**Weibull Bias**	0.020594	0.028155	0.037845	0.037867	0.038176	0.041955	0.042293	0.111293
	**T**	**S**	**P**	**Q**	**TW**	**M**	**MCD**	**TWR**
**Weibull RMSE**	0.111028	0.116633	0.156654	0.171996	0.174671	0.175598	0.176029	0.198387
	**n = 80 ρ = 0.2 Contamination = 10%**							
	**MCD**	**M**	**TW**	**TWR**	**Q**	**S**	**T**	**P**
**Normal Bias**	0.005091	0.011945	0.013318	0.016103	0.039726	0.06857	0.072424	0.135507
	**S**	**T**	**MCD**	**M**	**TW**	**Q**	**P**	**TWR**
**Normal RMSE**	0.130975	0.133047	0.154727	0.166011	0.166607	0.175882	0.176178	0.201692
	**TWR**	**M**	**TW**	**Q**	**T**	**MCD**	**S**	**P**
**Log-Normal Bias**	0.007237	0.031306	0.031327	0.045042	0.054922	0.060996	0.071641	0.148017
	**T**	**S**	**M**	**TW**	**MCD**	**Q**	**P**	**TWR**
**Log-Normal RMSE**	0.122246	0.132502	0.166284	0.166513	0.166756	0.175321	0.187102	0.200033
	**TWR**	**MCD**	**Q**	**TW**	**M**	**T**	**S**	**P**
**Weibull Bias**	0.00983	0.040277	0.04252	0.048285	0.048389	0.056793	0.07123	0.14103
	**T**	**S**	**MCD**	**M**	**TW**	**Q**	**P**	**TWR**
**Weibull RMSE**	0.122917	0.132397	0.166561	0.175862	0.175939	0.176303	0.180194	0.200706
	**n = 80 ρ = 0.5 Contamination = 0%**							
	**P**	**MCD**	**Q**	**T**	**TW**	**S**	**M**	**TWR**
**Normal Bias**	0.001713	0.007625	0.009058	0.015428	0.020571	0.022299	0.024009	0.1106
	**P**	**T**	**S**	**TW**	**M**	**Q**	**MCD**	**TWR**
**Normal RMSE**	0.084583	0.089477	0.092922	0.139803	0.142135	0.145882	0.147585	0.170307
	**Q**	**S**	**TW**	**M**	**T**	**P**	**MCD**	**TWR**
**Log-Normal Bias**	0.010055	0.02386	0.025194	0.02704	0.047948	0.056918	0.093371	0.109642
	**S**	**T**	**P**	**TW**	**M**	**Q**	**TWR**	**MCD**
**Log-Normal RMSE**	0.093051	0.102463	0.121112	0.13967	0.140423	0.146064	0.169549	0.175405
	**Q**	**M**	**TW**	**P**	**S**	**T**	**MCD**	**TWR**
**Weibull Bias**	0.01223	0.016173	0.016519	0.019275	0.023283	0.024105	0.048963	0.109607
	**S**	**T**	**P**	**TW**	**M**	**Q**	**MCD**	**TWR**
**Weibull RMSE**	0.093181	0.093301	0.09714	0.138204	0.138265	0.143133	0.166168	0.169562
	**n = 80 ρ = 0.5 Contamination = 5%**							
	**MCD**	**TW**	**M**	**Q**	**TWR**	**T**	**S**	**P**

**Normal Bias**	0.009927	0.023697	0.026548	0.057627	0.067754	0.102716	0.109515	0.272598
	**MCD**	**TW**	**M**	**T**	**S**	**TWR**	**Q**	**P**
**Normal RMSE**	0.134429	0.139918	0.140958	0.142807	0.148045	0.158438	0.161428	0.29179
	**TW**	**M**	**Q**	**TWR**	**MCD**	**T**	**S**	**P**
**Log-Normal Bias**	0.018936	0.020817	0.054488	0.068323	0.090349	0.095993	0.107459	0.297236
	**T**	**TW**	**M**	**S**	**TWR**	**Q**	**MCD**	**P**
**Log-Normal RMSE**	0.136236	0.139133	0.139498	0.146022	0.157579	0.161318	0.169239	0.316072
	**M**	**TW**	**MCD**	**Q**	**TWR**	**T**	**S**	**P**
**Weibull Bias**	0.021452	0.022794	0.049598	0.053728	0.070632	0.092136	0.107017	0.277992
	**T**	**M**	**TW**	**S**	**MCD**	**TWR**	**Q**	**P**
**Weibull RMSE**	0.13391	0.139023	0.139056	0.146169	0.156393	0.157626	0.158881	0.297388
		**n = 80**		**ρ = 0.5**		**Contamination = 10%**		
	**MCD**	**TW**	**M**	**TWR**	**Q**	**S**	**T**	**P**
**Normal Bias**	0.008386	0.010852	0.013671	0.020824	0.098793	0.175937	0.189366	0.346294
	**MCD**	**TW**	**M**	**TWR**	**Q**	**S**	**T**	**P**
**Normal RMSE**	0.127871	0.134439	0.136631	0.156011	0.183812	0.204691	0.216811	0.362931
	**TW**	**M**	**TWR**	**MCD**	**Q**	**T**	**S**	**P**
**Log-Normal Bias**	0.009766	0.011384	0.024171	0.090243	0.097651	0.15206	0.170115	0.360923
	**M**	**TW**	**TWR**	**MCD**	**T**	**Q**	**S**	**P**
**Log-Normal RMSE**	0.131235	0.131455	0.150739	0.163715	0.180153	0.180897	0.19787	0.376769
	**TWR**	**M**	**TW**	**MCD**	**Q**	**T**	**S**	**P**
**Weibull Bias**	0.022097	0.028574	0.029333	0.050542	0.099934	0.165386	0.172489	0.34863
	**TW**	**M**	**MCD**	**TWR**	**Q**	**T**	**S**	**P**
**Weibull RMSE**	0.134673	0.134705	0.147925	0.152772	0.181254	0.19393	0.200522	0.364767
		**n = 80**		**ρ = 0.7**		**Contamination = 0%**		
	**P**	**MCD**	**Q**	**T**	**TW**	**S**	**M**	**TWR**
**Normal Bias**	0.002087	0.00697	0.011164	0.016585	0.022235	0.023955	0.026255	0.100763
	**P**	**T**	**S**	**TW**	**M**	**MCD**	**Q**	**TWR**
**Normal RMSE**	0.058582	0.065249	0.070051	0.10106	0.103316	0.103956	0.111724	0.123608
	**Q**	**S**	**TW**	**M**	**T**	**P**	**MCD**	**TWR**
**Log-Normal Bias**	0.011248	0.02397	0.044412	0.046095	0.057271	0.061128	0.089638	0.100387
	**S**	**T**	**P**	**Q**	**TW**	**M**	**TWR**	**MCD**
**Log-Normal RMSE**	0.070151	0.089859	0.102009	0.112246	0.113052	0.11497	0.122982	0.144774
	**M**	**TW**	**Q**	**P**	**S**	**T**	**MCD**	**TWR**
**Weibull Bias**	0.004302	0.007119	0.014785	0.016767	0.02458	0.026875	0.047929	0.098608
	**P**	**S**	**T**	**TW**	**M**	**Q**	**TWR**	**MCD**
**Weibull RMSE**	0.069139	0.0699	0.07119	0.096395	0.097632	0.111686	0.121714	0.12748

	n = 80			ρ = 0.7		Contamination = 5%		
	**MCD**	**TW**	**M**	**TWR**	**Q**	**T**	**S**	**P**
**Normal Bias**	0.005583	0.024334	0.027444	0.062399	0.069848	0.139232	0.145448	0.379714
	**MCD**	**TW**	**M**	**TWR**	**Q**	**T**	**S**	**P**
**Normal RMSE**	0.091438	0.102124	0.104036	0.107097	0.139251	0.163149	0.168515	0.391306
	**TW**	**M**	**TWR**	**Q**	**MCD**	**T**	**S**	**P**
**Log-Normal Bias**	0.039242	0.041707	0.06281	0.070449	0.091104	0.130975	0.144816	0.406734
	**TWR**	**TW**	**M**	**Q**	**MCD**	**T**	**S**	**P**
**Log-Normal RMSE**	0.105	0.108574	0.110957	0.13933	0.141398	0.151713	0.166694	0.418571
	**M**	**TW**	**MCD**	**TWR**	**Q**	**T**	**S**	**P**
**Weibull Bias**	0.007854	0.00981	0.044205	0.063655	0.069856	0.128238	0.146369	0.389869
	**TW**	**M**	**TWR**	**MCD**	**Q**	**T**	**S**	**P**
**Weibull RMSE**	0.095863	0.096641	0.105274	0.117675	0.137945	0.150808	0.167806	0.400663
	**n = 80**			**ρ = 0.7**		**Contamination = 10%**		
	**MCD**	**TWR**	**TW**	**M**	**Q**	**S**	**T**	**P**
**Normal Bias**	0.004062	0.018041	0.019195	0.023109	0.128515	0.240084	0.262914	0.48255
	**MCD**	**TW**	**TWR**	**M**	**Q**	**S**	**T**	**P**
**Normal RMSE**	0.083157	0.097784	0.100271	0.100468	0.181402	0.257998	0.280978	0.493181
	**TWR**	**TW**	**M**	**MCD**	**Q**	**T**	**S**	**P**
**Log-Normal Bias**	0.016238	0.031396	0.034785	0.088608	0.12772	0.216973	0.239139	0.502018
	**TWR**	**TW**	**M**	**MCD**	**Q**	**T**	**S**	**P**
**Log-Normal RMSE**	0.101596	0.102579	0.105252	0.136281	0.181057	0.234246	0.256671	0.512646
	**M**	**TW**	**TWR**	**MCD**	**Q**	**T**	**S**	**P**
**Weibull Bias**	0.013865	0.015358	0.017902	0.041102	0.127506	0.23715	0.24014	0.488127
	**TW**	**M**	**TWR**	**MCD**	**Q**	**T**	**S**	**P**
**Weibull RMSE**	0.093524	0.093551	0.101484	0.109276	0.182294	0.255543	0.258067	0.498624
	**n = 80**			**ρ = 0.9**		**Contamination = 0%**		
	**P**	**MCD**	**T**	**Q**	**TW**	**S**	**M**	**TWR**
**Normal Bias**	0.000859	0.003447	0.009567	0.011203	0.013872	0.015017	0.017271	0.044101
	**P**	**T**	**S**	**MCD**	**TW**	**M**	**TWR**	**Q**
**Normal RMSE**	0.021516	0.026683	0.031352	0.039737	0.042026	0.045412	0.048276	0.057204
	**Q**	**S**	**TWR**	**TW**	**M**	**T**	**P**	**MCD**
**Log-Normal Bias**	0.010428	0.015417	0.043964	0.04593	0.047041	0.049068	0.056363	0.056746
	**S**	**TWR**	**Q**	**T**	**TW**	**P**	**M**	**MCD**
**Log-Normal RMSE**	0.031816	0.04823	0.05832	0.05954	0.066539	0.068522	0.06892	0.075824
	**TW**	**M**	**P**	**Q**	**S**	**T**	**MCD**	**TWR**
**Weibull Bias**	0.000845	0.001871	0.007496	0.011997	0.015244	0.016218	0.019318	0.044319
	**P**	**S**	**T**	**TW**	**M**	**TWR**	**MCD**	**Q**

**Weibull RMSE**	0.027884	0.032016	0.032869	0.037801	0.039715	0.04874	0.051435	0.059074
		**n = 80**		**ρ = 0.9**		**Contamination = 5%**		
	**MCD**	**TW**	**M**	**TWR**	**Q**	**T**	**S**	**P**
**Normal Bias**	0.002757	0.016172	0.019272	0.027902	0.064785	0.169098	0.172005	0.486646
	**MCD**	**TWR**	**TW**	**M**	**Q**	**T**	**S**	**P**
**Normal RMSE**	0.035098	0.038638	0.044596	0.047465	0.097113	0.183081	0.184019	0.493311
	**TWR**	**TW**	**M**	**MCD**	**Q**	**T**	**S**	**P**
**Log-Normal Bias**	0.027201	0.042559	0.044391	0.057596	0.066037	0.148101	0.17341	0.516125
	**TWR**	**TW**	**M**	**MCD**	**Q**	**T**	**S**	**P**
**Log-Normal RMSE**	0.038517	0.063716	0.066121	0.074881	0.09766	0.158367	0.186014	0.523309
	**TW**	**M**	**MCD**	**TWR**	**Q**	**T**	**S**	**P**
**Weibull Bias**	0.000573	0.002117	0.019815	0.027747	0.065688	0.152156	0.173822	0.490588
	**TW**	**TWR**	**M**	**MCD**	**Q**	**T**	**S**	**P**
**Weibull RMSE**	0.036988	0.038311	0.038611	0.049105	0.098036	0.164725	0.186047	0.497573
		**n = 80**		**ρ = 0.9**		**Contamination = 10%**		
	**MCD**	**TWR**	**TW**	**M**	**Q**	**S**	**T**	**P**
**Normal Bias**	0.001754	0.003764	0.01669	0.019465	0.127106	0.296339	0.331193	0.619754
	**MCD**	**TWR**	**TW**	**M**	**Q**	**S**	**T**	**P**
**Normal RMSE**	0.031535	0.037343	0.044956	0.047396	0.152965	0.308037	0.344608	0.627285
	**TWR**	**TW**	**M**	**MCD**	**Q**	**T**	**S**	**P**
**Log-Normal Bias**	0.003163	0.040061	0.043024	0.055607	0.126448	0.26232	0.29307	0.636495
	**TWR**	**TW**	**M**	**MCD**	**Q**	**T**	**S**	**P**
**Log-Normal RMSE**	0.037122	0.061079	0.064148	0.072568	0.153351	0.273798	0.304939	0.644178
	**TW**	**M**	**TWR**	**MCD**	**Q**	**S**	**T**	**P**
**Weibull Bias**	0.000059	0.001955	0.003795	0.018414	0.127555	0.298865	0.300308	0.622103
	**TW**	**TWR**	**M**	**MCD**	**Q**	**S**	**T**	**P**
**Weibull RMSE**	0.037641	0.038343	0.038687	0.045643	0.153734	0.310515	0.313265	0.629608
		**n = 160**		**ρ = 0**		**Contamination = 0%**		
	**P**	**T**	**S**	**Q**	**M**	**MCD**	**TW**	**TWR**
**Normal Bias**	0.000653	0.000824	0.000868	0.001745	0.001755	0.001894	0.001898	0.003406
	**T**	**P**	**S**	**Q**	**M**	**TW**	**MCD**	**TWR**
**Normal RMSE**	0.07885	0.079127	0.07916	0.122844	0.127144	0.127186	0.131212	0.150054
	**Q**	**P**	**S**	**TWR**	**T**	**MCD**	**TW**	**M**
**Log-Normal Bias**	0.000781	0.001108	0.001291	0.002992	0.015825	0.028326	0.045249	0.045597
	**P**	**S**	**T**	**MCD**	**Q**	**TW**	**M**	**TWR**
**Log-Normal RMSE**	0.078926	0.07957	0.080124	0.1219	0.125312	0.1351	0.135812	0.150606
	**Q**	**P**	**S**	**TWR**	**T**	**MCD**	**M**	**TW**

**Weibull Bias**	0.000194	0.000216	0.000462	0.000548	0.013436	0.034179	0.044282	0.044639
	**S**	**T**	**P**	**Q**	**MCD**	**TW**	**M**	**TWR**
**Weibull RMSE**	0.080489	0.080664	0.0809	0.124935	0.13455	0.137867	0.137897	0.152506
	**n = 160**			**ρ = 0**		**Contamination = 5%**		
	**P**	**MCD**	**S**	**T**	**TWR**	**Q**	**M**	**TW**
**Normal Bias**	0.001384	0.00144	0.001641	0.001713	0.001922	0.00258	0.007938	0.008266
	**S**	**T**	**P**	**MCD**	**Q**	**TW**	**M**	**TWR**
**Normal RMSE**	0.079092	0.07924	0.079833	0.117021	0.123116	0.12481	0.125562	0.149388
	**S**	**Q**	**P**	**TWR**	**T**	**MCD**	**TW**	**M**
**Log-Normal Bias**	0.000188	0.000401	0.00053	0.001515	0.012976	0.028432	0.049559	0.049981
	**P**	**S**	**T**	**MCD**	**Q**	**TW**	**M**	**TWR**
**Log-Normal RMSE**	0.078811	0.079191	0.079275	0.114353	0.123047	0.133599	0.134259	0.150122
	**Q**	**S**	**TWR**	**P**	**T**	**MCD**	**M**	**TW**
**Weibull Bias**	0.000196	0.000456	0.001006	0.001395	0.014515	0.03664	0.048111	0.048335
	**T**	**S**	**P**	**Q**	**MCD**	**M**	**TW**	**TWR**
**Weibull RMSE**	0.080552	0.080739	0.081118	0.122407	0.123749	0.137601	0.137856	0.15181
	**n = 160**			**ρ = 0**		**Contamination = 10%**		
	**P**	**MCD**	**S**	**TWR**	**T**	**Q**	**M**	**TW**
**Normal Bias**	0.000169	0.000302	0.000883	0.000924	0.001093	0.001558	0.028876	0.028938
	**T**	**S**	**P**	**MCD**	**Q**	**TW**	**M**	**TWR**
**Normal RMSE**	0.079636	0.079806	0.079985	0.108433	0.124411	0.126326	0.126641	0.150364
	**TWR**	**S**	**Q**	**P**	**T**	**MCD**	**M**	**TW**
**Log-Normal Bias**	0.000162	0.000484	0.000778	0.001947	0.015615	0.027098	0.062067	0.062383
	**P**	**S**	**T**	**MCD**	**Q**	**M**	**TW**	**TWR**
**Log-Normal RMSE**	0.078715	0.07944	0.08017	0.111622	0.123024	0.137878	0.138006	0.1496
	**P**	**S**	**TWR**	**Q**	**T**	**MCD**	**TW**	**M**
**Weibull Bias**	0	0.000583	0.001191	0.001703	0.02061	0.028363	0.067044	0.067237
	**S**	**P**	**T**	**MCD**	**Q**	**TW**	**M**	**TWR**
**Weibull RMSE**	0.078539	0.078992	0.080392	0.114472	0.123663	0.143616	0.143762	0.150638
	**n = 160**			**ρ = 0.2**		**Contamination = 0%**		
	**P**	**Q**	**MCD**	**TW**	**M**	**T**	**S**	**TWR**
**Normal Bias**	0.00097	0.002838	0.003059	0.006615	0.007168	0.007519	0.010651	0.065256
	**P**	**T**	**S**	**Q**	**TW**	**M**	**MCD**	**TWR**
**Normal RMSE**	0.075826	0.076541	0.077167	0.120321	0.124877	0.124938	0.127438	0.15342
	**Q**	**S**	**T**	**TW**	**M**	**P**	**MCD**	**TWR**
**Log-Normal Bias**	0.004454	0.009584	0.011574	0.017347	0.017357	0.030188	0.064937	0.065007
	**T**	**S**	**P**	**Q**	**TW**	**M**	**MCD**	**TWR**

**Log-Normal RMSE**	0.077139	0.077323	0.087459	0.121808	0.123677	0.124344	0.13566	0.154652
	**Q**	**T**	**S**	**P**	**M**	**TW**	**MCD**	**TWR**
**Weibull Bias**	0.00052	0.001085	0.008883	0.010256	0.038681	0.038948	0.051551	0.067452
	**T**	**S**	**P**	**Q**	**TW**	**M**	**MCD**	**TWR**
**Weibull RMSE**	0.07666	0.077603	0.080785	0.122497	0.131579	0.132294	0.142475	0.155345
		**n = 160**		**ρ = 0.2**		**Contamination = 5%**		
	**TW**	**M**	**MCD**	**Q**	**T**	**TWR**	**S**	**P**
**Normal Bias**	0.00162	0.002059	0.002545	0.023878	0.04129	0.042128	0.044377	0.108777
	**T**	**S**	**MCD**	**TW**	**M**	**Q**	**P**	**TWR**
**Normal RMSE**	0.087689	0.089262	0.111808	0.120606	0.121551	0.121953	0.134019	0.14881
	**Q**	**M**	**TW**	**T**	**TWR**	**S**	**MCD**	**P**
**Log-Normal Bias**	0.020973	0.022711	0.023183	0.03182	0.041826	0.04391	0.064968	0.122718
	**T**	**S**	**TW**	**Q**	**M**	**MCD**	**P**	**TWR**
**Log-Normal RMSE**	0.083058	0.089435	0.121134	0.121412	0.121527	0.128242	0.145989	0.146719
	**Q**	**T**	**TW**	**M**	**TWR**	**S**	**MCD**	**P**
**Weibull Bias**	0.022202	0.030226	0.039628	0.039649	0.04199	0.044427	0.051179	0.112718
	**T**	**S**	**Q**	**TW**	**M**	**MCD**	**P**	**TWR**
**Weibull RMSE**	0.082546	0.089143	0.12264	0.129583	0.130282	0.131156	0.138338	0.147498
		**n = 160**		**ρ = 0.2**		**Contamination = 10%**		
	**MCD**	**M**	**TW**	**TWR**	**Q**	**S**	**T**	**P**
**Normal Bias**	0.009381	0.012559	0.012771	0.012959	0.041888	0.071636	0.075198	0.137907
	**MCD**	**S**	**T**	**TW**	**M**	**Q**	**TWR**	**P**
**Normal RMSE**	0.106182	0.106797	0.109303	0.119959	0.120668	0.129942	0.146352	0.158652
	**TWR**	**M**	**TW**	**Q**	**T**	**MCD**	**S**	**P**
**Log-Normal Bias**	0.012869	0.031821	0.032221	0.042015	0.053865	0.065251	0.069747	0.147226
	**T**	**S**	**M**	**TW**	**MCD**	**Q**	**TWR**	**P**
**Log-Normal RMSE**	0.09422	0.104821	0.121717	0.121997	0.127996	0.128574	0.144226	0.167104
	**TWR**	**Q**	**MCD**	**M**	**T**	**TW**	**S**	**P**
**Weibull Bias**	0.016231	0.040967	0.047214	0.056509	0.056529	0.056585	0.070698	0.142473
	**T**	**S**	**MCD**	**Q**	**TW**	**M**	**TWR**	**P**
**Weibull RMSE**	0.095258	0.105405	0.121871	0.126438	0.132284	0.132674	0.143057	0.162752
		**n = 160**		**ρ = 0.5**		**Contamination = 0%**		
	**P**	**MCD**	**Q**	**TW**	**M**	**T**	**S**	**TWR**
**Normal Bias**	0.000538	0.003049	0.003315	0.009939	0.011151	0.012993	0.019408	0.121494
	**P**	**T**	**S**	**TW**	**M**	**MCD**	**Q**	**TWR**
**Normal RMSE**	0.059878	0.063372	0.066233	0.098413	0.099383	0.099474	0.100795	0.150825
	**Q**	**TW**	**M**	**S**	**T**	**P**	**MCD**	**TWR**

**Log-Normal Bias**	0.005296	0.016927	0.017281	0.019827	0.044076	0.058341	0.101259	0.120757
	**S**	**T**	**P**	**TW**	**M**	**Q**	**MCD**	**TWR**
**Log-Normal RMSE**	0.066035	0.077701	0.096019	0.098364	0.098845	0.102591	0.144744	0.150078
	**Q**	**P**	**S**	**T**	**M**	**TW**	**MCD**	**TWR**
**Weibull Bias**	0.004384	0.018531	0.020955	0.020995	0.02864	0.029231	0.060427	0.120601
	**S**	**T**	**P**	**TW**	**M**	**Q**	**MCD**	**TWR**
**Weibull RMSE**	0.066721	0.067182	0.069695	0.101273	0.101539	0.101594	0.125738	0.149464
		**n = 160**		**ρ = 0 .5**		**Contamination = 5%**		
	**MCD**	**TW**	**M**	**Q**	**TWR**	**T**	**S**	**P**
**Normal Bias**	0.0027	0.009218	0.010455	0.04947	0.080413	0.098391	0.104863	0.269609
	**MCD**	**TW**	**M**	**Q**	**T**	**S**	**TWR**	**P**
**Normal RMSE**	0.088757	0.096878	0.097399	0.113991	0.11988	0.125431	0.125778	0.279005
	**TW**	**M**	**Q**	**TWR**	**T**	**MCD**	**S**	**P**
**Log-Normal Bias**	0.009301	0.010636	0.05111	0.078565	0.093833	0.095368	0.105214	0.297409
	**M**	**TW**	**T**	**Q**	**TWR**	**S**	**MCD**	**P**
**Log-Normal RMSE**	0.097672	0.097678	0.115162	0.117614	0.125254	0.125574	0.138509	0.307276
	**TW**	**M**	**Q**	**MCD**	**TWR**	**T**	**S**	**P**
**Weibull Bias**	0.035939	0.035965	0.048674	0.054389	0.083883	0.087756	0.103466	0.277685
	**M**	**TW**	**T**	**Q**	**MCD**	**S**	**TWR**	**P**
**Weibull RMSE**	0.102234	0.102279	0.1104	0.11472	0.115146	0.123774	0.127678	0.287118
		**n = 160**		**ρ = 0.5**		**Contamination = 10%**		
	**TW**	**M**	**MCD**	**TWR**	**Q**	**S**	**T**	**P**
**Normal Bias**	0.004131	0.005406	0.006189	0.030739	0.095218	0.172771	0.186352	0.343281
	**MCD**	**TW**	**M**	**TWR**	**Q**	**S**	**T**	**P**
**Normal RMSE**	0.082082	0.095804	0.096309	0.109941	0.143783	0.187349	0.200443	0.351216
	**TW**	**M**	**TWR**	**MCD**	**Q**	**T**	**S**	**P**
**Log-Normal Bias**	0.004921	0.00566	0.031574	0.092056	0.092115	0.149697	0.168974	0.359451
	**M**	**TW**	**TWR**	**MCD**	**Q**	**T**	**S**	**P**
**Log-Normal RMSE**	0.095717	0.095884	0.111308	0.133367	0.141499	0.16489	0.18386	0.367761
	**TWR**	**TW**	**M**	**MCD**	**Q**	**T**	**S**	**P**
**Weibull Bias**	0.031301	0.039165	0.03968	0.055715	0.094557	0.16484	0.171694	0.349123
	**TW**	**M**	**MCD**	**TWR**	**Q**	**T**	**S**	**P**
**Weibull RMSE**	0.102525	0.102928	0.111887	0.112015	0.143993	0.179803	0.18632	0.357357
		**n = 160**		**ρ = 0.7**		**Contamination = 0%**		
	**P**	**MCD**	**Q**	**TW**	**T**	**M**	**S**	**TWR**
**Normal Bias**	0.001956	0.003256	0.006457	0.01284	0.014637	0.014726	0.021552	0.106051
	**P**	**T**	**S**	**MCD**	**TW**	**M**	**Q**	**TWR**

**Normal RMSE**	0.041293	0.046813	0.051144	0.068907	0.069471	0.070927	0.077147	0.116637
	**Q**	**S**	**M**	**TW**	**T**	**P**	**MCD**	**TWR**
**Log-Normal Bias**	0.007247	0.021249	0.035065	0.035204	0.055263	0.065848	0.097235	0.106696
	**S**	**T**	**Q**	**TW**	**M**	**P**	**TWR**	**MCD**
**Log-Normal RMSE**	0.050503	0.073506	0.078089	0.07909	0.07964	0.088782	0.117126	0.125136
	**Q**	**P**	**M**	**S**	**TW**	**T**	**MCD**	**TWR**
**Weibull Bias**	0.006341	0.015252	0.020538	0.020607	0.021227	0.023601	0.049049	0.106649
	**P**	**S**	**T**	**M**	**TW**	**Q**	**MCD**	**TWR**
**Weibull RMSE**	0.050038	0.05018	0.052089	0.069588	0.069801	0.080425	0.095359	0.117005
	**n = 160**			**ρ = 0.7**		**Contamination = 5%**		
	**MCD**	**TW**	**M**	**Q**	**TWR**	**T**	**S**	**P**
**Normal Bias**	0.00282	0.01216	0.013588	0.062557	0.072428	0.135643	0.141855	0.377541
	**MCD**	**TW**	**M**	**TWR**	**Q**	**T**	**S**	**P**
**Normal RMSE**	0.059798	0.068619	0.069369	0.091789	0.104524	0.147981	0.153499	0.383281
	**TW**	**M**	**Q**	**TWR**	**MCD**	**T**	**S**	**P**
**Log-Normal Bias**	0.027412	0.028921	0.06295	0.072734	0.09298	0.124182	0.139315	0.406702
	**TW**	**M**	**TWR**	**Q**	**MCD**	**T**	**S**	**P**
**Log-Normal RMSE**	0.074792	0.076204	0.092297	0.105041	0.119269	0.135365	0.150708	0.412409
	**M**	**TW**	**MCD**	**Q**	**TWR**	**T**	**S**	**P**
**Weibull Bias**	0.023223	0.023554	0.048358	0.062171	0.07268	0.123066	0.141049	0.385252
	**TW**	**M**	**MCD**	**TWR**	**Q**	**T**	**S**	**P**
**Weibull RMSE**	0.067862	0.068383	0.090277	0.092306	0.105632	0.13564	0.152699	0.390823
	**n = 160**			**ρ = 0.7**		**Contamination = 10%**		
	**MCD**	**TW**	**M**	**TWR**	**Q**	**S**	**T**	**P**
**Normal Bias**	0.002642	0.009861	0.011177	0.026883	0.121225	0.236408	0.259098	0.479505
	**MCD**	**TW**	**M**	**TWR**	**Q**	**S**	**T**	**P**
**Normal RMSE**	0.055492	0.067955	0.06897	0.072588	0.15106	0.245565	0.268412	0.484913
	**TW**	**M**	**TWR**	**MCD**	**Q**	**T**	**S**	**P**
**Log-Normal Bias**	0.023134	0.025521	0.025831	0.092216	0.121295	0.209657	0.233985	0.500086
	**TW**	**TWR**	**M**	**MCD**	**Q**	**T**	**S**	**P**
**Log-Normal RMSE**	0.072896	0.073335	0.073918	0.118974	0.151883	0.218835	0.243036	0.505482
	**M**	**TW**	**TWR**	**MCD**	**Q**	**T**	**S**	**P**
**Weibull Bias**	0.026889	0.027482	0.029502	0.044936	0.118849	0.23118	0.233462	0.483345
	**M**	**TW**	**TWR**	**MCD**	**Q**	**T**	**S**	**P**
**Weibull RMSE**	0.067863	0.068333	0.072463	0.084347	0.148224	0.240657	0.242603	0.488767
	**n = 160**			**ρ = 0.9**		**Contamination = 0%**		
	**P**	**MCD**	**Q**	**TW**	**T**	**M**	**S**	**TWR**

**Normal Bias**	0.000405	0.001465	0.005296	0.007155	0.007445	0.008758	0.011656	0.04668
	**P**	**T**	**S**	**MCD**	**TW**	**M**	**Q**	**TWR**
**Normal RMSE**	0.015243	0.01915	0.022452	0.025694	0.027997	0.02933	0.039867	0.048582
	**Q**	**S**	**M**	**TW**	**T**	**TWR**	**MCD**	**P**
**Log-Normal Bias**	0.004855	0.011507	0.040276	0.040523	0.045679	0.046702	0.058611	0.061985
	**S**	**Q**	**TWR**	**T**	**TW**	**M**	**MCD**	**P**
**Log-Normal RMSE**	0.022498	0.039376	0.048588	0.051474	0.052702	0.053317	0.067715	0.070201
	**Q**	**M**	**P**	**TW**	**S**	**T**	**MCD**	**TWR**
**Weibull Bias**	0.005884	0.007067	0.007086	0.008021	0.012019	0.013844	0.021103	0.046624
	**P**	**S**	**T**	**TW**	**M**	**MCD**	**Q**	**TWR**
**Weibull RMSE**	0.020029	0.022718	0.023994	0.026116	0.026191	0.039711	0.040287	0.048557
		**n = 160**		**ρ = 0.9**		**Contamination = 5%**		
	**MCD**	**TW**	**M**	**TWR**	**Q**	**T**	**S**	**P**
**Normal Bias**	0.001249	0.008182	0.009382	0.031388	0.059488	0.16825	0.171041	0.486868
	**MCD**	**TW**	**M**	**TWR**	**Q**	**T**	**S**	**P**
**Normal RMSE**	0.022324	0.027705	0.028642	0.035862	0.077262	0.175302	0.177192	0.490202
	**TWR**	**TW**	**M**	**MCD**	**Q**	**T**	**S**	**P**
**Log-Normal Bias**	0.031738	0.037746	0.039041	0.05838	0.059948	0.141455	0.168285	0.51377
	**TWR**	**TW**	**M**	**MCD**	**Q**	**T**	**S**	**P**
**Log-Normal RMSE**	0.036307	0.050076	0.051827	0.067314	0.078517	0.146655	0.174631	0.517329
	**M**	**TW**	**MCD**	**TWR**	**Q**	**T**	**S**	**P**
**Weibull Bias**	0.006672	0.007748	0.021336	0.031455	0.058926	0.14821	0.169966	0.488783
	**TW**	**M**	**TWR**	**MCD**	**Q**	**T**	**S**	**P**
**Weibull RMSE**	0.025989	0.026366	0.036213	0.037915	0.07706	0.154661	0.176112	0.492091
		**n = 160**		**ρ = 0.9**		**Contamination = 10%**		
	**MCD**	**TW**	**TWR**	**M**	**Q**	**S**	**T**	**P**
**Normal Bias**	0.001214	0.008789	0.009279	0.010211	0.120468	0.292656	0.328179	0.617445
	**MCD**	**TWR**	**TW**	**M**	**Q**	**S**	**T**	**P**
**Normal RMSE**	0.021057	0.025992	0.028527	0.029725	0.135151	0.298819	0.335236	0.621249
	**TWR**	**TW**	**M**	**MCD**	**Q**	**T**	**S**	**P**
**Log-Normal Bias**	0.008627	0.034668	0.036998	0.056884	0.120286	0.255748	0.289889	0.636016
	**TWR**	**TW**	**M**	**MCD**	**Q**	**T**	**S**	**P**
**Log-Normal RMSE**	0.026167	0.046797	0.048985	0.065798	0.134789	0.261665	0.295944	0.639812
	**M**	**TW**	**TWR**	**MCD**	**Q**	**S**	**T**	**P**
**Weibull Bias**	0.007294	0.008062	0.009963	0.019489	0.120238	0.290978	0.293041	0.618144
	**TW**	**M**	**TWR**	**MCD**	**Q**	**S**	**T**	**P**
**Weibull RMSE**	0.025863	0.025973	0.026142	0.034989	0.135008	0.297123	0.299763	0.621803

	n = 320 ρ = 0 Contamination = 0%							
	**S**	**P**	**T**	**MCD**	**Q**	**TWR**	**M**	**TW**
**Normal Bias**	0.000004	0.000036	0.000053	0.000552	0.000684	0.001095	0.001168	0.001178
	**T**	**P**	**S**	**Q**	**TW**	**M**	**MCD**	**TWR**
**Normal RMSE**	0.055539	0.055551	0.05564	0.086862	0.090455	0.090634	0.090849	0.106954
	**P**	**S**	**TWR**	**Q**	**T**	**MCD**	**TW**	**M**
**Log-Normal Bias**	0.000112	0.000244	0.000442	0.000726	0.015303	0.03469	0.045889	0.046012
	**S**	**P**	**T**	**Q**	**MCD**	**TW**	**M**	**TWR**
**Log-Normal RMSE**	0.05567	0.05621	0.057411	0.088826	0.089167	0.101264	0.101597	0.107784
	**TWR**	**S**	**P**	**Q**	**T**	**MCD**	**M**	**TW**
**Weibull Bias**	0.001298	0.001311	0.001578	0.002344	0.015739	0.042551	0.046625	0.04693
	**P**	**S**	**T**	**Q**	**MCD**	**TW**	**M**	**TWR**
**Weibull RMSE**	0.056308	0.056492	0.058341	0.08828	0.102595	0.105684	0.105974	0.109366
	**n = 320 ρ = 0 Contamination = 5%**							
	**Q**	**MCD**	**T**	**S**	**P**	**TWR**	**M**	**TW**
**Normal Bias**	0.000312	0.000477	0.000597	0.000886	0.001182	0.001492	0.005987	0.006216
	**T**	**S**	**P**	**MCD**	**Q**	**TW**	**M**	**TWR**
**Normal RMSE**	0.055676	0.055819	0.056083	0.079373	0.087521	0.089766	0.089772	0.108274
	**Q**	**S**	**TWR**	**P**	**T**	**MCD**	**M**	**TW**
**Log-Normal Bias**	0.00006	0.000592	0.000677	0.000727	0.013481	0.032068	0.051938	0.052144
	**P**	**S**	**T**	**MCD**	**Q**	**TW**	**M**	**TWR**
**Log-Normal RMSE**	0.055672	0.055901	0.057212	0.084566	0.087721	0.104414	0.104455	0.108819
	**S**	**TWR**	**Q**	**P**	**T**	**MCD**	**M**	**TW**
**Weibull Bias**	0.000053	0.000298	0.000537	0.000668	0.015995	0.03901	0.052998	0.053199
	**S**	**P**	**T**	**Q**	**MCD**	**TWR**	**TW**	**M**
**Weibull RMSE**	0.055978	0.056375	0.057621	0.087101	0.093152	0.104762	0.105879	0.106276
	**n = 320 ρ = 0 Contamination = 10%**							
	**S**	**Q**	**MCD**	**P**	**TWR**	**T**	**M**	**TW**
**Normal Bias**	0.000001	0.000123	0.000695	0.000855	0.001131	0.001896	0.02762	0.027838
	**T**	**S**	**P**	**MCD**	**Q**	**TW**	**M**	**TWR**
**Normal RMSE**	0.055005	0.055078	0.055456	0.074986	0.086479	0.091121	0.091203	0.108004
	**P**	**Q**	**S**	**TWR**	**T**	**MCD**	**TW**	**M**
**Log-Normal Bias**	0.00054	0.00075	0.001156	0.001759	0.01556	0.030951	0.061155	0.061253
	**S**	**P**	**T**	**MCD**	**Q**	**TW**	**M**	**TWR**
**Log-Normal RMSE**	0.056173	0.05632	0.057512	0.081047	0.088329	0.107181	0.107448	0.109476
	**TWR**	**Q**	**S**	**P**	**T**	**MCD**	**M**	**TW**
**Weibull Bias**	0.000011	0.000233	0.000301	0.000302	0.020081	0.035019	0.067013	0.067127
	**S**	**P**	**T**	**MCD**	**Q**	**TWR**	**TW**	**M**

**Weibull RMSE**	0.054976	0.056028	0.057746	0.085629	0.087702	0.106177	0.113402	0.11353
	**n = 320**			**ρ = 0.2**		**Contamination = 0%**		
	**P**	**Q**	**MCD**	**TW**	**M**	**T**	**S**	**TWR**
**Normal Bias**	0.000028	0.000953	0.001127	0.002894	0.003128	0.005878	0.009026	0.068907
	**P**	**T**	**S**	**Q**	**MCD**	**M**	**TW**	**TWR**
**Normal RMSE**	0.053845	0.054494	0.05507	0.085451	0.087649	0.088361	0.08876	0.121114
	**Q**	**S**	**T**	**TW**	**M**	**P**	**TWR**	**MCD**
**Log-Normal Bias**	0.002438	0.009358	0.011575	0.020005	0.020503	0.031527	0.068649	0.071977
	**S**	**T**	**P**	**Q**	**TW**	**M**	**MCD**	**TWR**
**Log-Normal RMSE**	0.055153	0.055476	0.067707	0.085972	0.089615	0.08989	0.110187	0.121479
	**T**	**Q**	**S**	**P**	**TW**	**M**	**MCD**	**TWR**
**Weibull Bias**	0.001647	0.001902	0.009615	0.01182	0.04213	0.04235	0.058276	0.068554
	**T**	**S**	**P**	**Q**	**TW**	**M**	**MCD**	**TWR**
**Weibull RMSE**	0.054506	0.055637	0.057848	0.086411	0.099369	0.099696	0.111667	0.121119
	**n = 320**			**ρ = 0.2**		**Contamination = 5%**		
	**MCD**	**TW**	**M**	**Q**	**T**	**S**	**TWR**	**P**
**Normal Bias**	0.000954	0.002552	0.002902	0.019204	0.039862	0.042731	0.04686	0.108252
	**T**	**S**	**MCD**	**TW**	**M**	**Q**	**TWR**	**P**
**Normal RMSE**	0.067031	0.068916	0.076328	0.08593	0.08642	0.087709	0.110268	0.121209
	**Q**	**TW**	**M**	**T**	**S**	**TWR**	**MCD**	**P**
**Log-Normal Bias**	0.021402	0.026633	0.026833	0.030745	0.043321	0.043451	0.070313	0.122052
	**T**	**S**	**Q**	**TW**	**M**	**MCD**	**TWR**	**P**
**Log-Normal RMSE**	0.062101	0.069878	0.088655	0.089071	0.089456	0.105114	0.10991	0.134842
	**Q**	**T**	**S**	**TW**	**M**	**TWR**	**MCD**	**P**
**Weibull Bias**	0.019451	0.027233	0.042075	0.045445	0.045864	0.045871	0.055902	0.111042
	**T**	**S**	**Q**	**TW**	**M**	**MCD**	**TWR**	**P**
**Weibull RMSE**	0.060467	0.068875	0.087056	0.100404	0.100627	0.102225	0.111289	0.124152
	**n = 320**			**ρ = 0.2**		**Contamination = 10%**		
	**MCD**	**TW**	**M**	**TWR**	**Q**	**S**	**T**	**P**
**Normal Bias**	0.005516	0.01726	0.017344	0.020024	0.038944	0.06936	0.073229	0.13659
	**MCD**	**TW**	**M**	**S**	**T**	**Q**	**TWR**	**P**
**Normal RMSE**	0.07134	0.086247	0.08658	0.088207	0.091353	0.092477	0.103275	0.147111
	**TWR**	**TW**	**M**	**Q**	**T**	**MCD**	**S**	**P**
**Log-Normal Bias**	0.018471	0.036574	0.036895	0.039677	0.051749	0.064789	0.068015	0.145732
	**T**	**S**	**TW**	**M**	**Q**	**MCD**	**TWR**	**P**
**Log-Normal RMSE**	0.074256	0.086955	0.092966	0.093069	0.093224	0.100905	0.103329	0.156273
	**TWR**	**Q**	**MCD**	**T**	**TW**	**M**	**S**	**P**

**Weibull Bias**	0.019303	0.039049	0.051901	0.054406	0.059123	0.059756	0.068554	0.140514
	**T**	**S**	**MCD**	**Q**	**TW**	**M**	**TWR**	**P**
**Weibull RMSE**	0.077148	0.088565	0.094024	0.095314	0.104122	0.104589	0.105692	0.151264
	**n = 320**			**ρ = 0.5**		**Contamination = 0%**		
	**P**	**MCD**	**Q**	**TW**	**M**	**T**	**S**	**TWR**
**Normal Bias**	0.000526	0.001168	0.002608	0.005406	0.005797	0.012324	0.018596	0.125454
	**P**	**T**	**S**	**MCD**	**M**	**TW**	**Q**	**TWR**
**Normal RMSE**	0.041982	0.04561	0.048515	0.067935	0.069502	0.06987	0.072312	0.14024
	**Q**	**M**	**TW**	**S**	**T**	**P**	**MCD**	**TWR**
**Log-Normal Bias**	0.001011	0.011542	0.011982	0.018411	0.042974	0.061201	0.10469	0.126386
	**S**	**T**	**TW**	**M**	**Q**	**P**	**MCD**	**TWR**
**Log-Normal RMSE**	0.047793	0.062201	0.068882	0.069501	0.070104	0.083424	0.127458	0.140248
	**Q**	**P**	**S**	**T**	**TW**	**M**	**MCD**	**TWR**
**Weibull Bias**	0.003329	0.017831	0.019077	0.019083	0.034862	0.035187	0.068092	0.124374
	**S**	**T**	**P**	**Q**	**TW**	**M**	**MCD**	**TWR**
**Weibull RMSE**	0.048629	0.048907	0.05067	0.072061	0.077281	0.077501	0.105182	0.139415
	**n = 320**			**ρ = 0.5**		**Contamination = 5%**		
	**MCD**	**M**	**TW**	**Q**	**TWR**	**T**	**S**	**P**
**Normal Bias**	0.002163	0.004407	0.004567	0.047937	0.08523	0.098368	0.104378	0.269045
	**MCD**	**TW**	**M**	**Q**	**T**	**TWR**	**S**	**P**
**Normal RMSE**	0.060631	0.06886	0.069174	0.089487	0.109627	0.109687	0.115249	0.273854
	**TW**	**M**	**Q**	**TWR**	**T**	**MCD**	**S**	**P**
**Log-Normal Bias**	0.004525	0.004588	0.046114	0.085757	0.089868	0.101544	0.102544	0.29699
	**TW**	**M**	**Q**	**T**	**TWR**	**S**	**MCD**	**P**
**Log-Normal RMSE**	0.066872	0.066941	0.085664	0.100946	0.10889	0.112885	0.123181	0.301772
	**TW**	**M**	**Q**	**MCD**	**TWR**	**T**	**S**	**P**
**Weibull Bias**	0.039831	0.040223	0.046862	0.06339	0.085275	0.088103	0.10414	0.277871
	**TW**	**M**	**Q**	**MCD**	**T**	**TWR**	**S**	**P**
**Weibull RMSE**	0.07852	0.07878	0.087795	0.096188	0.100077	0.109378	0.114709	0.282666
	**n = 320**			**ρ = 0.5**		**Contamination = 10%**		
	**MCD**	**M**	**TW**	**TWR**	**Q**	**S**	**T**	**P**
**Normal Bias**	0.001727	0.003441	0.003678	0.037728	0.092362	0.17045	0.184241	0.341933
	**MCD**	**TW**	**M**	**TWR**	**Q**	**S**	**T**	**P**
**Normal RMSE**	0.056522	0.068542	0.068751	0.084419	0.120128	0.178093	0.19157	0.346057
	**TW**	**M**	**TWR**	**Q**	**MCD**	**T**	**S**	**P**
**Log-Normal Bias**	0.000179	0.000776	0.036207	0.092163	0.097176	0.147236	0.167872	0.360434
	**TW**	**M**	**TWR**	**MCD**	**Q**	**T**	**S**	**P**

**Log-Normal RMSE**	0.067287	0.067382	0.081963	0.118393	0.119194	0.155074	0.175368	0.364466
	**TWR**	**TW**	**M**	**MCD**	**Q**	**T**	**S**	**P**
**Weibull Bias**	0.035017	0.044546	0.045021	0.061373	0.093503	0.164469	0.170943	0.348565
	**TW**	**M**	**TWR**	**MCD**	**Q**	**T**	**S**	**P**
**Weibull RMSE**	0.079961	0.080192	0.082494	0.091489	0.120929	0.172412	0.178706	0.352656
	**n = 320**			**ρ = 0.7**		**Contamination = 0%**		
	**P**	**MCD**	**Q**	**TW**	**M**	**T**	**S**	**TWR**
**Normal Bias**	0.000768	0.001586	0.00388	0.006445	0.006896	0.012513	0.019124	0.110739
	**P**	**T**	**S**	**MCD**	**TW**	**M**	**Q**	**TWR**
**Normal RMSE**	0.029036	0.033661	0.037702	0.04714	0.048213	0.048782	0.054885	0.11558
	**Q**	**S**	**M**	**TW**	**T**	**P**	**MCD**	**TWR**
**Log-Normal Bias**	0.002684	0.018857	0.027793	0.028455	0.052663	0.067179	0.098247	0.11119
	**S**	**Q**	**TW**	**M**	**T**	**P**	**MCD**	**TWR**
**Log-Normal RMSE**	0.037478	0.054616	0.057007	0.057381	0.062889	0.080925	0.112632	0.116058
	**Q**	**P**	**S**	**T**	**TW**	**M**	**MCD**	**TWR**
**Weibull Bias**	0.004598	0.015472	0.019232	0.022244	0.027675	0.027915	0.054609	0.110676
	**P**	**S**	**T**	**TW**	**M**	**Q**	**MCD**	**TWR**
**Weibull RMSE**	0.037301	0.037712	0.039678	0.053399	0.053642	0.055032	0.080114	0.115579
	**n = 320**			**ρ = 0.7**		**Contamination = 5%**		
	**MCD**	**TW**	**M**	**Q**	**TWR**	**T**	**S**	**P**
**Normal Bias**	0.001543	0.00729	0.007511	0.0594	0.077004	0.134156	0.140068	0.377329
	**MCD**	**TW**	**M**	**Q**	**TWR**	**T**	**S**	**P**
**Normal RMSE**	0.040044	0.047089	0.047193	0.084155	0.086149	0.140379	0.146006	0.380215
	**TW**	**M**	**Q**	**TWR**	**MCD**	**T**	**S**	**P**
**Log-Normal Bias**	0.022646	0.023124	0.060041	0.076104	0.096211	0.122074	0.139015	0.409879
	**TW**	**M**	**Q**	**TWR**	**MCD**	**T**	**S**	**P**
**Log-Normal RMSE**	0.053512	0.054085	0.085219	0.085754	0.109772	0.127966	0.144959	0.412967
	**TW**	**M**	**MCD**	**Q**	**TWR**	**T**	**S**	**P**
**Weibull Bias**	0.028136	0.028137	0.052834	0.059865	0.075966	0.121668	0.139738	0.384203
	**M**	**TW**	**MCD**	**Q**	**TWR**	**T**	**S**	**P**
**Weibull RMSE**	0.054072	0.054268	0.075375	0.084875	0.085664	0.128177	0.145784	0.387126
	**n = 320**			**ρ = 0.7**		**Contamination = 10%**		
	**MCD**	**M**	**TW**	**TWR**	**Q**	**S**	**T**	**P**
**Normal Bias**	0.000678	0.003724	0.00374	0.03295	0.117287	0.234226	0.257467	0.480345
	**MCD**	**M**	**TW**	**TWR**	**Q**	**S**	**T**	**P**
**Normal RMSE**	0.037582	0.046713	0.046879	0.057386	0.133864	0.23879	0.262092	0.48297
	**TW**	**M**	**TWR**	**MCD**	**Q**	**T**	**S**	**P**

**Log-Normal Bias**	0.019201	0.020778	0.031655	0.094105	0.11846	0.204541	0.231136	0.500073
	**TW**	**M**	**TWR**	**MCD**	**Q**	**T**	**S**	**P**
**Log-Normal RMSE**	0.050686	0.051523	0.056131	0.107369	0.134278	0.20911	0.235798	0.502753
	**TWR**	**TW**	**M**	**MCD**	**Q**	**T**	**S**	**P**
**Weibull Bias**	0.032685	0.033152	0.03352	0.048018	0.116959	0.231028	0.232731	0.483933
	**TW**	**M**	**TWR**	**MCD**	**Q**	**T**	**S**	**P**
**Weibull RMSE**	0.055814	0.055931	0.057755	0.069634	0.133091	0.23589	0.237432	0.486639
		**n = 320**		**ρ = 0.9**		**Contamination = 0%**		
	**P**	**MCD**	**Q**	**TW**	**M**	**T**	**S**	**TWR**
**Normal Bias**	0.000143	0.000527	0.00279	0.003697	0.004176	0.006364	0.010026	0.048068
	**P**	**T**	**S**	**MCD**	**TW**	**M**	**Q**	**TWR**
**Normal RMSE**	0.010889	0.013977	0.016864	0.017658	0.018862	0.019153	0.027773	0.048948
	**Q**	**S**	**M**	**TW**	**T**	**TWR**	**MCD**	**P**
**Log-Normal Bias**	0.002251	0.010064	0.037537	0.03812	0.044483	0.048148	0.060435	0.066476
	**S**	**Q**	**M**	**TW**	**T**	**TWR**	**MCD**	**P**
**Log-Normal RMSE**	0.016817	0.027364	0.044476	0.044723	0.047379	0.049029	0.065261	0.071703
	**Q**	**P**	**S**	**M**	**TW**	**T**	**MCD**	**TWR**
**Weibull Bias**	0.003051	0.006555	0.010261	0.011507	0.011779	0.012309	0.023871	0.048062
	**P**	**S**	**T**	**M**	**TW**	**Q**	**MCD**	**TWR**
**Weibull RMSE**	0.0146	0.016826	0.018434	0.020331	0.02045	0.027304	0.034072	0.048918
		**n = 320**		**ρ = 0.9**		**Contamination = 5%**		
	**MCD**	**TW**	**M**	**TWR**	**Q**	**T**	**S**	**P**
**Normal Bias**	0.000658	0.004655	0.005093	0.033462	0.056515	0.16671	0.168748	0.48551
	**MCD**	**TW**	**M**	**TWR**	**Q**	**T**	**S**	**P**
**Normal RMSE**	0.015279	0.018825	0.019155	0.035556	0.066173	0.170411	0.17193	0.48723
	**TWR**	**TW**	**M**	**Q**	**MCD**	**T**	**S**	**P**
**Log-Normal Bias**	0.033371	0.034711	0.035805	0.056116	0.059657	0.138283	0.165958	0.515527
	**TWR**	**TW**	**M**	**MCD**	**Q**	**T**	**S**	**P**
**Log-Normal RMSE**	0.035443	0.04131	0.042753	0.064172	0.066044	0.140903	0.169145	0.517349
	**M**	**TW**	**MCD**	**TWR**	**Q**	**T**	**S**	**P**
**Weibull Bias**	0.010762	0.011049	0.022681	0.033452	0.056114	0.145974	0.168039	0.487298
	**M**	**TW**	**MCD**	**TWR**	**Q**	**T**	**S**	**P**
**Weibull RMSE**	0.019724	0.019805	0.031539	0.035489	0.066276	0.149223	0.171182	0.489004
		**n = 320**		**ρ = 0.9**		**Contamination = 10%**		
	**MCD**	**TW**	**M**	**TWR**	**Q**	**S**	**T**	**P**
**Normal Bias**	0.000254	0.004511	0.004886	0.012272	0.117108	0.290101	0.325983	0.615026
	**MCD**	**TW**	**M**	**TWR**	**Q**	**S**	**T**	**P**

**Normal RMSE**	0.014308	0.018766	0.019012	0.020752	0.124418	0.293102	0.329472	0.616878
	**TWR**	**TW**	**M**	**MCD**	**Q**	**T**	**S**	**P**
**Log-Normal Bias**	0.011867	0.03097	0.03306	0.05747	0.116109	0.249324	0.287382	0.636944
	**TWR**	**TW**	**M**	**MCD**	**Q**	**T**	**S**	**P**
**Log-Normal RMSE**	0.020357	0.03768	0.039923	0.061891	0.123516	0.252351	0.290407	0.638836
	**M**	**TW**	**TWR**	**MCD**	**Q**	**S**	**T**	**P**
**Weibull Bias**	0.011262	0.011348	0.012338	0.020817	0.115685	0.289939	0.292526	0.617365
	**M**	**TW**	**TWR**	**MCD**	**Q**	**S**	**T**	**P**
**Weibull RMSE**	0.019997	0.020002	0.020546	0.028949	0.123099	0.292989	0.295958	0.619204
